# Functions of the Thyroid-Stimulating Hormone on Key Developmental Features Revealed in a Series of Zebrafish Dyshormonogenesis Models

**DOI:** 10.3390/cells10081984

**Published:** 2021-08-04

**Authors:** Jia Song, Yao Lu, Xiaoxia Cheng, Chuang Shi, Qiyong Lou, Xia Jin, Jiangyan He, Gang Zhai, Zhan Yin

**Affiliations:** 1 State Key Laboratory of Freshwater Ecology and Biotechnology, Institute of Hydrobiology, Chinese Academy of Sciences, Wuhan 430072, China; songjia@ihb.ac.cn (J.S.); luyao@ihb.ac.cn (Y.L.); shichuang@ihb.ac.cn (C.S.); louqiyong@ihb.ac.cn (Q.L.); jinxia@ihb.ac.cn (X.J.); jyhe@ihb.ac.cn (J.H.); 2 College of Advanced Agricultural Sciences, University of Chinese Academy of Sciences, Beijing 100049, China; 3 Key Laboratory of Receptors-Mediated Gene Regulation and Drug Discovery, School of Basic Medical Sciences, Henan University, Kaifeng 475004, China; 10190130@vip.henu.edu.cn

**Keywords:** thyroid-stimulating hormone, thyroglobulin, *slc16a2*, larval-to-juvenile transition, goiter, secondary sex characteristic

## Abstract

The hypothalamic–pituitary–thyroid (HPT) axis regulates many critical features in vertebrates. Utilizing TALENs and CRISPR/Cas9 techniques, thyroid-stimulating hormone subunit beta a (*tshba*), thyroglobulin (*tg*), and solute carrier family 16 member 2 (*slc16a2*) mutant zebrafish lines were generated. Among the three mutants, the earliest time point for the significantly altered T3 contents was observed in *tshba* mutants, which resulted in the most severe defects, including typical defects such as the retardation of inflated anterior swimming bladder (aSB), proper formation of fin ray and posterior squamation (SP), the larval-to-juvenile transition (LTJT) process, juvenile growth retardation, and mating failure. In *tg* mutants, which are actually compensated with an alternative splicing form, growth retardation was observed in the juvenile stage without LTJT and reproductive defects. The evident goiter phenotype was only observed in *tg*- and *slc16a2* mutants, but not in *tshba* mutants. Other than goiters being observed, no other significant developmental defects were found in the *slc16a2* mutants. Regarding the reproductive defects observed in *tshba* mutants, the defective formation of the secondary sex characteristics (SSCs) was observed, while no obvious alterations during gonad development were found. Based on our analyses, zebrafish at the 6–12 mm standard length or 16–35 days post-fertilization (dpf) should be considered to be in their LTJT phase. Using a series of zebrafish dyshormonogenesis models, this study demonstrated that the TSH function is critical for the proper promotion of zebrafish LTJT and SSC formation. In addition, the elevation of TSH levels appears to be essential for goiter appearance in zebrafish.

## 1. Introduction

The thyroid hormone (TH) controls different processes in vertebrates, including metamorphosis, oxidative metabolism, and annual change of the photoperiod [1,2,3,4]. Although the specifics may differ, the mechanisms and compositions of the hypothalamic–pituitary–thyroid (HPT) axis in vertebrates are relatively conserved. Thyroid hormonogenesis is regulated via thyrotropin-releasing hormone (TRH) and thyroid-stimulating hormone (TSH), and circulating triiodothyronine (T3) and thyroxine (T4) can act as dynamic negative feedback signals for the hypothalamus and pituitary to regulate the levels of TH via the production of TRH and TSH. Therefore, TSH, a heterodimer protein of two glycoprotein hormone subunits α and β, both of which are secreted from the anterior lobe of the pituitary gland, is a major signal that plays a positive regulatory role in thyroid homeostasis. The α subunit is common to the three pituitary glycoprotein hormones, whereas the β subunit is TSH-specific, providing receptor specificity [5,6]. In thyroid follicular cells, thyroglobulin (TG) is a large dimeric protein that is required for TH synthesis, storage, and secretion processes. THs, including T4 and T3, have iodine atoms. After TH synthesis in the thyroid gland, solute carrier family 16 member 2 (SLC16A2) (also named monocarboxylate transporter 8) is a specific TH transporter in certain tissues that allows the cellular entry of THs to access their nuclear receptors for downstream TH signaling activation.

Nearly 10% of the human population may be at risk of developing a thyroid-related disease during their lifetime, such as central congenital hypothyroidism (CCH), goiter, and Allan-Herndon-Dudley syndrome (AHDS) [7,8,9]. Certain types of mutations of the tshb, *tshr*, *tg*, *slc16a2*, *solute carrier 5a5* (*slc5a5*), *dual oxidase 2* (*duox2*) genes, etc., have been reported to be associated with these congenital diseases [9,10,11,12,13,14,15]. To allow the investigation of the pathophysiological mechanisms of various types of these disorders, several animal models have been generated. With convenient targeted genetic procedures, zebrafish have emerged as a fascinating vertebrate animal model for biomedical studies. The AA sequence of Tshb in zebrafish is highly similar to that of the mammalian TSHβ molecule, especially at its critical domains [16]. However, the role of TSH in teleost fish, especially during post-embryonic stages, remains uncertain due to the lack of a relevant genetic model. TG is a large dimeric glycoprotein required for TH synthesis in vertebrate thyrocytes. The reported human monomer TG molecular mass is 330 kDa, containing nearly 2750 AA residues, comprising type I, II, and III repeat motif-containing regions and the C-terminal cholinesterase-like domain [17]. Patients and rodent models with *tg* gene mutations typically develop hypothyroidism goiters [18,19]. However, it is possible that certain weak genetic variations within the *tg* locus contribute to euthyroid familial goiters. Beside mammals, available Tg sequences have been identified in teleost genomes; for example, a putative zebrafish Tg contains 2733 AAs reported previously [20]. Zebrafish Tg levels are commonly used as an indicator for endogenous TH synthesis activity [21]. However, the physiological importance of fish Tg protein is yet to be experimentally investigated. Patients with *SLC16A2* mutations exhibit psychomotor deficits associated with Allan–Herndon–Dudley syndrome (AHDS) [8], with rare cases also exhibiting goiter, while no apparent brain development and neurological defects have been observed in *Slc16a2* mutant mice [22,23]. Intriguingly, mutations in the zebrafish *slc16a2* gene cause neurological and behavioral phenotypical changes in larvae, which makes zebrafish an emerging model for exploring the process of TH signaling in the presence of psychomotor defects. However, little information is currently available regarding the syndromes of the *slc16a2* mutant zebrafish during their adult stage, including goiter [24]. Several other homozygous gene-deficient germline zebrafish models related to TH signaling have been established, including *deiodinase type 2* (*dio2*), *organic anion-transporting polypeptide 1c1* (*oatp1c1*, also known as *slco1c1*), *dual oxidase* (*duox*), and *thyroid hormone receptor a* and *b* (*thra* and *thrb*). Like the *slc16a2* mutant model, *oatp1c1* deficiency causes neural alterations and hyperactivity of the HPT axis activity in mutant larvae and adults. Goiter has been observed in *oatp1c1*- and *slc16a2*-deficient zebrafish [25]. NADPH oxidase DUOX generates hydrogen peroxide, which is a crucial electron acceptor during thyroid peroxidase-catalyzed iodination and coupling reactions during the TH synthesis process. DUOX-deficient zebrafish exhibit observable phenotypes reminiscent of human CCH, including growth retardation and goiter. Besides, delayed initiation of anterior swim bladder (aSB) development, one of the major features in larval-to-juvenile transition (LTJT) in zebrafish, has also been observed in *duox*-deficient zebrafish [26]. Most recently, studies on the deficiency of Thra in zebrafish indicate that decreased TH levels and impaired post-larval growth and skin development occur in *thrab* mutant zebrafish [27].

Here, *tshb*, *tg*, and *slc16a2* mutant zebrafish models were generated using TALENs and CRISPR/Cas9 techniques. The observable changes in dynamics of whole-body TH contents were recorded in all three mutant zebrafish types and compared with those of their control fish siblings. In particular, significantly decreased TH levels were observed 25 days post-fertilization (dpf) in *tshb* mutant zebrafish, and 60 dpf in *tg* mutant zebrafish. Interestingly, impaired development of the features of LTJT and reproductive activities in zebrafish was only found in the *tshba* mutants, which is relevant to the early initiation of the dyshormonogenesis observed at the larval stage in *tshba* mutants. Both *tshba* and *tg* mutant fish displayed growth retardation with various extensions during the juvenile stage initially. They eventually reached their normal size during their adult stage. In addition, adulthood goiter phenotypes were only observed in *tg* and *slc16a2* mutant zebrafish, indicating the critical role of TSH in thyrocyte proliferation and its involvement in the negative regulatory network through endogenous TH levels. These mutant zebrafish provided a series of teleost models for understanding the regulatory functions of TH signaling in animal development further.

## 2. Materials and Methods

### 2.1. Zebrafish Maintenance and Treatment

All zebrafish (AB strain and *tshba*, *tg*, and *slc16a2* mutants generated using AB strain) were maintained in a circulating water system with a 14 h light and 10 h dark cycle at 28.5 °C and were fed twice daily with newly hatched brine shrimp (*Artemia salina*). The procedure for fish maintenance followed that of a previous study [28]. All fish experiments were conducted in accordance with the Guiding Principles for the Care and Use of Laboratory Animals and were approved by the Institute of Hydrobiology, Chinese Academy of Sciences (Approval ID: IHB2013724).

For T3 treatment, the crossed progeny of *tshba* heterozygote mutants were used. Larvae were reared in a 1 L tank at a density of 30 larvae per tank. The water in each 1 L tank was replaced daily. Stock solutions of T3 (T2877, Sigma-Aldrich, St Louis, MO, USA) were initially dissolved in dimethyl sulfoxide (DMSO) then diluted in water for the experimental treatments. The final DMSO concentrations were below 0.1%. Larvae were treated with 2.5 μg/L of T3 for rescue, starting at 12 dpf. The control larvae were exposed to DMSO. T3-treated larvae were sampled and analyzed at different stages.

### 2.2. Establishment of Mutant Lines

The *tshba* mutant line was obtained using the TALEN gene targeting technique as previously described [29]. Specific TALEN target sites were designed and located on the third exon of the zebrafish *tshba* gene. Paired TALENs were constructed with the Golden Gate TALEN Kit [30]. TALEN mRNAs (100 ng/µL) were microinjected into the one-cell stage wild-type zebrafish embryos. For *tshba* mutation screening, the target genomic regions were amplified by PCR reaction with gene-specific primers and digested with BtsCI restriction enzymes, which is located within the targeting region of *tshba*. The digested PCR products were separated by electrophoresis using a 1% agarose gel.

The *tg* and *slc16a2* mutant lines were generated using the CRISPR/Cas9 system as previously described [31]. Specific guide RNA (gRNA) target sites located at the fourth exon of the *tg* gene (*tg* gRNA sequences: 5′-GACTCAGGTGAGTACCAGCAGG-3′) and first exon of the *slc16a2* gene (*slc16a2* gRNA sequences: 5′-GGAGTCCTCTTCCCCTGCG GAGG-3′) were selected, and gRNA was transcribed with the TranscriptAid T7 High-Yield Transcription Kit (Thermo Fisher Scientific, Waltham, MA, USA). For Cas9 messenger RNA (mRNA) synthesis, pXT7-humanized Cas9 expression vector was linearized with XbaI restriction enzymes, purified, and transcribed using mMESSAGE mMACHINE T7 Ultra Kit (Ambion, Carlsbad, CA, USA) according to the manufacturer’s instructions. A mixture of Cas9 mRNA (300 ng/µL) and gRNA (20 ng/µL) was then co-injected into one-cell stage zebrafish embryos. A PAGE-based genotyping assay was conducted to examine the mutation of the *tg* or *slc16a2* gene as previously described [32]. Briefly, the target genomic regions of each individual were amplified by a standard PCR reaction with gene-specific primers. The PCR products were denatured at 95 °C for 10 min to form heteroduplexes and were then loaded into a 10% polyacrylamide gel with the running condition of 220 V for 1.5 h.

To obtain germline mutations of *tshba*, *tg*, and *slc16a2* genes, the F0 generation zebrafish were raised to adulthood and mated with wild-type fish to generate heterozygous F1 offspring. The heterozygous F1 generation fish were genotyped via the assay mentioned above and confirmed by sequencing. The individuals with frame-shift sequence alterations were selected. Males and females of the F1 generation carrying the same mutation were mated to produce F2 homozygous mutants, which were genotyped via BstCI digestion or PAGE assay. The specific primers used for PCR genotyping are listed in Supplementary Table S1.

### 2.3. RNA Extraction and qPCR

Total RNA was extracted using TRIzol reagent, and cDNA was synthesized using an oligo (dT) 18 primer and a RevertAid First Strand cDNA Synthesis Kit (Thermo Fisher Scientific, Rockford, IL, USA) according to the manufacturer’s instructions. The gene-specific qPCR primers were designed using the National Center for Biotechnology Information (NCBI) primer BLAST service and are listed in Supplementary Table S1. All mRNA levels were calculated as fold-expression levels relative to the housekeeping gene *eukaryotic translation elongation factor 1 alpha*. Each sample was run in triplicate, and the results were expressed according to a previously described method [33]. The primers for qPCR (Supplementary Table S1) were validated by DNA sequencing and agarose gel electrophoresis of the PCR products. The qPCR was performed using the TransStart TIP Green qPCR SuperMix Kit (AQ141, Transgene Biotech, Beijing, China) according to the manufacturer’s instructions. qPCR cycling conditions were: 95 °C for 5 min [95 °C for 15 s, 60 °C for 15 s, 72 °C for 15 s] (45 cycles), 95 °C for 60 s, followed by dissociation curve analysis.

### 2.4. Whole-Mount Immunofluorescence

Whole-mount immunofluorescence was carried out as described previously [26,34]. Briefly, more than 60 zebrafish larvae at 5 dpf derived from the mating of heterozygous *tshba* parents after treatment, were incubated at 4 °C in blocking buffer containing the primary antibody against T4 (1:1000 dilution, polyclonal rabbit anti T4, 08658501, MP Biomedicals, LCC, Illkirch, France). After several wash steps in PBST containing 1% BSA, larvae were then incubated overnight at 4 °C in blocking buffer containing the secondary antibodies (1:250 dilution, goat anti-rabbit IgG-FITC, SA00001, Proteintech Group, Chicago, IL, USA). Fluorescent images were taken of all the stained larvae. Next, the larvae were washed in PBST and subjected to genotyping. Based on the results of genotyping, all images from the homozygous *tshba* mutants and wild-type larvae were grouped for fluorescent intensity quantification. For each embryo, the ventral view of the heads was obtained following a previously described protocol [26,34] using ImageJ software (National Institutes of Health, Bethesda, MD, USA). Integrated density was selected as the parameter for measuring fluorescence inside the thyroid follicles, which was conducted using the “Integrated Density” tool of ImageJ software.

### 2.5. Protein Extraction and Western Blot Analysis

For Western blots, the heads were dissected from the 5 dpf zebrafish larvae. Heads from 12–15 larvae were pooled and homogenized in lysis buffer containing proteinase inhibitors (1%, *v*/*v*), a phosphatase inhibitor (0.1%, *v*/*v*), and phenylmethanesulfonyl fluoride (1%, *v*/*v*). Protein concentrations were measured by the Bradford method [35]. Approximately 40 μg of each protein sample were loaded into a Tris-glycine 10% polyacrylamide gel. After electrophoresis, proteins were electro-transferred onto nitrocellulose membranes. Membranes were probed with the primary antibodies against TG (polyclonal rabbit anti-human TG, IR509, Dako, Carpinteria, CA, USA) overnight at 4 °C and incubated with HRP-conjugated secondary antibodies at 37 °C for 2 h. After several wash steps in Tris-buffered saline Tween 20, the blots were developed using an ECL reagent (P90720, Millipore, Billerica, MA, USA). Using a rabbit anti-human TG serum antibody, Western blotting detected a single protein of ∼260 kDa for TG. Bands on autoradiographs were quantified by densitometry.

### 2.6. Analyses of Growth and Developmental Timing

The crossed progeny of the heterozygote mutants were used for analyses of postembryonic growth and development. Fish from the crossed clutch were isolated at 10 dpf and reared individually in water-filled plastic cups, the water of which was changed daily. Each individual was anesthetized briefly with tricaine (MS222) and was immersed in 1% methylcellulose. Fish were imaged daily from 10 to 30 dpf and every three days from 33 to 60 dpf using an Olympus SZX-16 epifluorescence stereomicroscope (Olympus Corp., Tokyo, Japan). Images were further processed to evaluate post-embryonic growth and development. Each fish was genotyped at 60 dpf. To analyze post-embryonic growth, images were measured in ImageJ for standard length (SL) and staged according to external anatomical features [36]. Differences in the age and SL between wild-type and mutants upon reaching defined stages of post-embryonic development were analyzed by ANOVAs to test the effects of genotypes, developmental stages, and phenotypes on the two groups.

### 2.7. Thyroid Hormone Quantification

Since the size of zebrafish is small, the levels of T3 and T4 in the whole body were quantified with the liquid chromatography-quadrupole time-of-flight mass spectrometry (LC-MS/MS) method as described previously [37]. Three groups of fish were sampled at different stages of development. The fish were genotyped individually with the genomic DNA of the tail fin. The numbers of individuals used in each group for the time points of 16, 20, 25, 30, and 60 dpf were 40, 20, 8, 4, and 2 fish as a sample, respectively. The triplicate samples were then pooled in pre-weighed microcentrifuge tubes and wet weights were recorded. The samples were stored at −80 °C for the quantification of TH levels. TH was extracted from the samples then analyzed by UPLC-MS/MS (Xevo TQ-S Triple Quadrupole Mass Spectrometry, Waters, Milford, MA, USA), following a previously described method [38].

### 2.8. Behavioral Assays

Behavioral tests were conducted using a ZebraBox system (ViewPoint Life Sciences, Lyon, France). To measure swimming ability, each test fish was placed into a separate Petri dish with 10 mL of water. Twelve fish were tested from each group. The swimming behavior tests were recorded for 10 min from the dorsal viewpoint of the dish. The resulting data were analyzed using ZebraBox software (Viewpoint Life Science, Lyon, France). To measure mating behavior, the mating process in a spawning tank was recorded. The videos started 5 min after removing the separators. The duration of the video for each pair was 5 min. The mating behaviors were analyzed using ZebraBox software.

### 2.9. Micro-CT Scan

The fish at the 30 dpf stage were euthanized with MS222 then fixed overnight in 4% PFA at 4 °C. The samples were scanned under X-ray with micro-CT visualization according to a previously described method. Briefly, the fixed sample was placed in a sample holder and observed using a micro-CT (SkyScan 1276 micro-CT system, Bruker, Kontich, Belgium) with an X-ray source of 42 kV and a 200 μA current. A high-resolution camera was used with a 0.4–0.7 °C rotation pitch and a 3.0 µm image resolution. In total, 1027 projection images were recorded within 10 min of exposure time. Images were reconstructed and analyzed with CTvox software provided by the manufacturer.

### 2.10. Statistical Analysis

Detailed information regarding the number of zebrafish used in each experiment has been provided in all figure legends. All analyses were performed using GraphPad Prism 5.0 software (GraphPad Software Inc., San Diego, CA, USA), and the differences were assessed using Student’s *t*-test. The results are expressed as the mean ± SEM. For all statistical comparisons, *p* < 0.05 was used to indicate a statistically significant difference.

## 3. Results

### 3.1. Generation of Tshba Mutant Zebrafish

**Figure 1:**
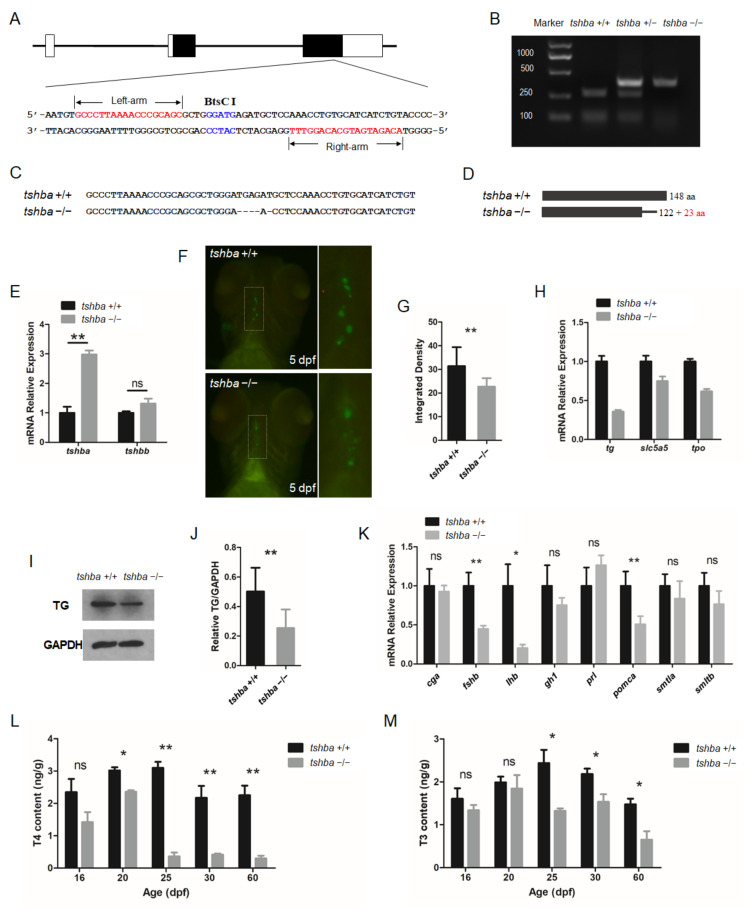
Generation of the *tshba* mutant zebrafish. (**A**) Schematic representation of the genomic structure of *tshba* and the targeting site on exon 3. The regions of the left and right arms are labeled above and below, respectively. The BtsCI site in the spacer is labeled above. (**B**) Genotyping of the *tshba* +/+, *tshba* +/−, and *tshba* −/− individuals represented with the un-cleaved and cleaved PCR products is indicated. (**C**) DNA sequences of the targeting regions of the *tshba* locus in wild-type and *tshba* mutant zebrafish. (**D**) Predicted putative wild-type and mutant Tshba protein structures. The 5 bp deletion generated a miscoded peptide after the 122th amino acid (AA) with the 23 miscoded AAs at the C-terminus. (**E**) Comparison results of quantitative RT-PCR analyses of the *tshba* and *tshbb* genes between the *tshba* mutant and its wild-type siblings at 5 dpf. (**F**) T4 immunofluorescence analyses at 5 dpf. The thyroid follicles are labeled in green for T4 with thyroxine immunofluorescence. Details of T4 immunofluorescent signals in the dotted white box are shown at high magnification. (**G**) Quantitative analyses of the T4 immunofluorescence signal at 5 dpf. Measurements were taken from wild-type zebrafish (n = 10) and *tshba* mutants (n = 9). (**H**) Quantitative RT-PCR analyses of TH synthesis-related genes, including the *tg*, *slc5a5*, and *tpo* gene in the *tshba* mutant and their wild-type siblings at 5 dpf. (**I**) Representative Western blotting of the TG protein from zebrafish at the 2 mpf stage using anti-TG antibodies. (**J**) Quantification of the relative expression of TG protein of the Western blot analyses. Measurements were taken from wild-type zebrafish (n = 5) and *tshba* mutants (n = 5). (**K**) Quantitative RT-PCR analyses of pituitary hormone genes, including *cga*, *fshb*, *lhb*, *gh1*, *prl*, *pomca*, *smtla*, and *smtlb*, in the *tshba* mutant and its wild-type sibling at 20 dpf. (**L**,**M**) Whole-body T4 levels (**L**) and T3 levels (**M**) in zebrafish at various stages. Measurements of THs were performed with three groups of wild-type zebrafish and three groups of *tshba* mutant zebrafish at 16, 20, 25, and 60 dpf stages. * *p* < 0.05; ** *p* < 0.01, ns: No significance.

In zebrafish, TSHβ was encoded by the *tshba* gene located at chromosome 6. To investigate the potential functions of *tshba* in zebrafish, the zebrafish *tshba* locus was targeted using the TALENs technique. Mutant fish with a 5 bp deletion in the third exon of the *tshba* gene of the *tshba* locus was achieved (Figure 1A).

The F2 progenies were screened using BtsCI restriction enzymes. The BtsCI digestion assay revealed that the fragments containing target sites of wild-type fish were completely cut into two smaller fragments, those of heterozygotes were cut partly, and those of homozygotes were not cut at all (Figure 1B). To confirm the mutations in the target region, PCR-amplified cDNAs of homozygous mutants were sequenced. The results showed that the sequence of the *tshba* mutant gene carried a 5-nucleotide deletion and a single-nucleotide substitution (G→C) on the *tshba* exon 3 (Figure 1C). The cDNA carrying the 5 bp deletion led to a putative premature termination of the Tshb protein at AA position 145, with 23 AAs different from the wild-type Tshb protein sequence at the C-terminus (Figure 1D).

The zebrafish TSHβa protein was compared with the protein sequence of human TSHβ protein and *tshb* C105V patients were identified that exhibited severe syndromes due to the last conserved cysteine being lost in the mutant human TSHβ protein, and damaged seat-belt structure [9] (Supplementary Figure S1A). The multi-alignment analysis of the TSHβa proteins indicated the high similarity between our zebrafish *tshba* mutation and the human tshb C105C mutation. There were significant discrepancies between the sequences of the zebrafish TSHba and TSHbb proteins (Supplementary Figure S1B). Additionally, in *tshba* mutants, significantly elevated *tshba* mRNA levels were found, but no compensative *tshbb* mRNA levels were observed, compared with those in the control fish (Figure 1B). All whole mount immunofluorescence experiments were conducted with 81 larvae at the 5 dpf stage using T4 antibodies, which indicated that the immunoreactivity for T4 levels in *tshba* mutant larvae (n = 20) was considerably less than that in their wild-type siblings (n = 21) (Figure 1F,G). The mRNA expression levels of the key genes involved in TH synthesis in larvae at 5 dpf were tested with quantitative RT-PCR. The results showed that the expression levels of *tg*, *thyroid peroxidase* (*tpo*), and *slc5a5* genes were significantly downregulated in *tshba* mutant larvae compared to those in their wild-type siblings (Figure 1H). Western blot analysis showed that the level of TG protein was significantly downregulated in *tshba* mutant fish (Figure 1I,J). In terms of the impacts on pituitary hormone secretion, the expression levels of the *fshb*, *lhb*, and *pomca* genes were significantly downregulated in the *tsha* mutant zebrafish at the 20 dpf stage, compared with those in their wild-type siblings (Figure 1K).

To investigate the effect of the Tshba mutation on thyroid hormonogenesis in zebrafish, we measured the total TH content at different developmental stages using the LC-MS/MS method. There were no significant differences in either T4 or T3 contents between *tshba* mutants and their wild-type siblings before the 16 dpf stage (Figure 1L,M). However, significantly decreased T4 content in *tshba* mutants at 20 dpf was observed, while the T3 content was not affected at this stage. The significant differences in both T4 and T3 contents between *tshba* mutants and their control siblings were increasingly evident after the 25 dpf stage, with T4 depletions being more severe than T3 ones. These results suggest that the *tshba* mutation causes impairment of the dynamic endogenous TH production, which serves as a model for the fish CCH model.

### 3.2. Growth Performance of the Tshba Mutant Zebrafish

**Figure 2:**
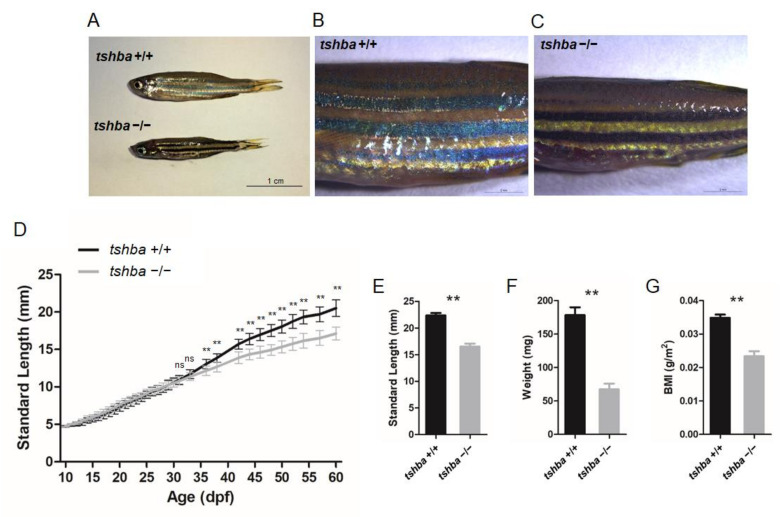
Somatic growth features of the *tshba* mutant fish and control wild-type siblings. (**A**) Wild-type control fish and *tshba* mutant zebrafish at 2 mpf. (**B**,**C**) Pigment pattern of wild-type control fish (**B**) and *tshba* mutant fish (**C**) at 2 mpf. (**D**) Curves of the somatic growth of wild-type control fish and *tshba* mutant fish. Means of standard length (SL) are presented as the growth curve. Measurements were performed with wild-type fish (n = 10) and *tshba* mutants (n = 10). Significantly distinguishable differences beginning at 36 dpf. (**E**–**G**) SL (**E**), whole-body weight (**F**), and body mass index (**G**) of *tshba* mutant fish and their wild-type siblings at 2 mpf. Measurements were taken of wild-type fish (n = 12) and *tshba* mutant fish (n = 10). ** *p* < 0.01.

An apparent dwarfism phenotype in adult *tshba* mutant fish was recorded only after the 35 dpf stage (Figure 2A).

In addition to this apparent phenotype, the altered pigment pattern has been observed in homozygous *tshba* mutants, the pigmentation of which was darker than that of their wild-type siblings at the two months post-fertilization (mpf) stage (Figure 2B,C). By measuring the body lengths and weights, it was found that the bodies of the *tshba* mutant fish were significantly shorter and less heavy than those of the control group, with a significant reduction in their body mass index (BMI) (Figure 2E–G).

To determine the exact stage when the growth phenotype of the *tshba* mutant fish can be distinguished from that of control fish, we measured the body length of wild-type and *tshba* mutant individuals from 10 to 60 dpf. The growth curves were drawn using the average SL data from 10 homozygous *tshba* mutants and 11 wild-type siblings. The results showed no significant differences between the sizes of *tshba* mutant fish and their wild-type siblings until 35 dpf (Figure 2D). From the 35 dpf onward, the size differences between the *tshba* mutants and the controls became increasingly apparent with age.

### 3.3. Post-Embryonic Development of Tshba Mutant Zebrafish

**Figure 3:**
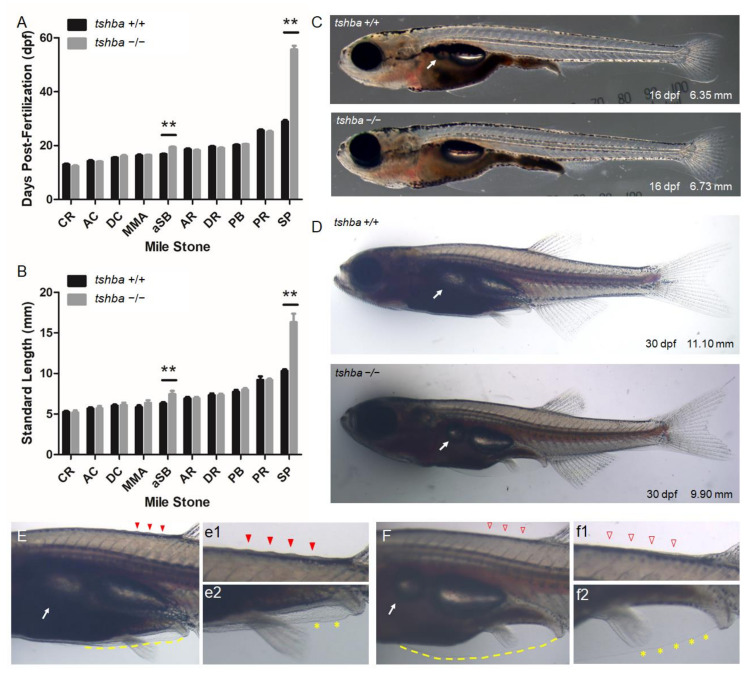
Comparative morphological analyses between wild-type fish and tshba mutant fish prior to the juvenile stage. (**A**) Comparative morphological features and their relationships with age of fish between *tshba* mutant zebrafish and their wild-type siblings. (**B**) Comparative morphological features observed and their relationships with fish size between *tshba* mutant zebrafish and wild-type siblings. The x-axes show developmental milestones describing early larval stages (left) toward late larval stages (right). Measurements were taken of wild-type fish (n = 11) and *tshba* mutants (n = 10). (**C**) Morphological features of wild-type and *tshba* mutants at 16 dpf. Arrows indicate the appearance of inflated anterior swim bladders. (**D**) Morphological features of wild-type and *tshba* mutants at 30 dpf. Arrows indicate the appearance of inflated aSBs. (**E**) Higher magnification views of wild-type fish (30 dpf) showing an inflated aSB (arrows), anterior squamation (SA; details in (**e1**), red arrowhead), minor fin fold (dotted yellow line, details in (**e2**) labelled with yellow asterisks), vent, and anal fin. (**F**) Higher magnification views of *tshba* mutant fish (30 dpf) showing a smaller inflated aSB (arrows), SA (details in (**f1**), red arrowhead), minor fin fold (dotted yellow line, details in (**f2**) labeled with yellow asterisks), vent, and anal fin. Stage abbreviations: CR, caudal fin ray appearance; AC, anal fin condensation; DC, dorsal fin condensation; MMA, metamorphic melanophore appearance; aSB, inflation of anterior swim bladder lobe; AR, anal fin ray appearance; DR, dorsal fin ray appearance; PB, pelvic fin bud appearance; PR, pelvic fin ray appearance; SP, posterior squamation; SA, anterior squamation.** *p* < 0.01.

To further characterize *tshba* mutant fish during post-embryonic developmental progression, we thoroughly assessed the morphological features during the post-embryonic stages following the methods of previous studies [36,39]. We found that the period of aSB inflation and scale appearance (SP) were obviously delayed in *tshba* mutant fish compared to that in their wild-type counterparts (Figure 3A,B), while other stage-specific milestones described previously appeared normal in both *tshba* mutant and wild-type fish with no significant differences (Figure 3A,B).

Detailed observations on the relationships between the developmental stage and age revealed that *tshba* mutant fish took a longer time to reach the stage of proper aSB (16.23 ± 1.52 dpf for wild-type vs. 20.71 ± 2.34 dpf for *tshba* mutant) and SP (28.92 ± 1.98 dpf for wild-type vs. 55.70 ± 4.42 dpf for *tshba* mutant) formation compared to their wild-type siblings (Figure 3A). Correspondingly, the bodies of *tshba* mutant fish were significantly longer when they reached aSB (6.24 ± 0.16 mm for wild-type vs. 7.78 ± 0.80 mm for *tshba* mutant) and SP (10.31 ± 0.20 mm for wild-type vs. 16.33 ± 1.04 mm for *tshba* mutant) formation stages, compared to those of their wild-type siblings (Figure 3B). In addition, while wild-type fish completed the anterior squamation process around the 30 dpf stage, most *tshba* mutant fish did not develop their scales anterior to the dorsal fin by 60 dpf (six of the ten mutant fish, data not shown).

We focused on the morphological features at 16 and 30 dpf as these periods represent the average ages at which wild-type fish reach the aSB and SP formation stages, respectively. By 16 dpf, the wild-type larvae had an inflated aSB, while none of the *tshba* mutant larvae had an aSB (Figure 3C). By the 30 dpf stage, a relatively larger aSB compared to the posterior swim chamber was seen in wild-type larvae, while in *tshba* mutant larvae, a relatively smaller inflated aSB was observed (Figure 3D). Among 15 *tshba* mutant larvae, one larva did not develop an inflated aSB at 30 dpf (data not shown). In wild-type fish at 30 dpf, scales were apparent anterior to the dorsal fin, as evidenced by ridges (Figure 3E). In contrast, none of the *tshba* mutant fish of the same age developed scales (Figure 3F). In addition, when only small remnants of the pelvic fin fold were present ventrally in wild-type fish (Figure 3E,e2), the pelvic fin fold of *tshba* mutant fish was much more apparent and remained to be resorbed (Figure 3F,f2).

### 3.4. Rescue Effects of the Supplemental Exogenous T3 Treatments on Tshba Mutants

**Figure 4:**
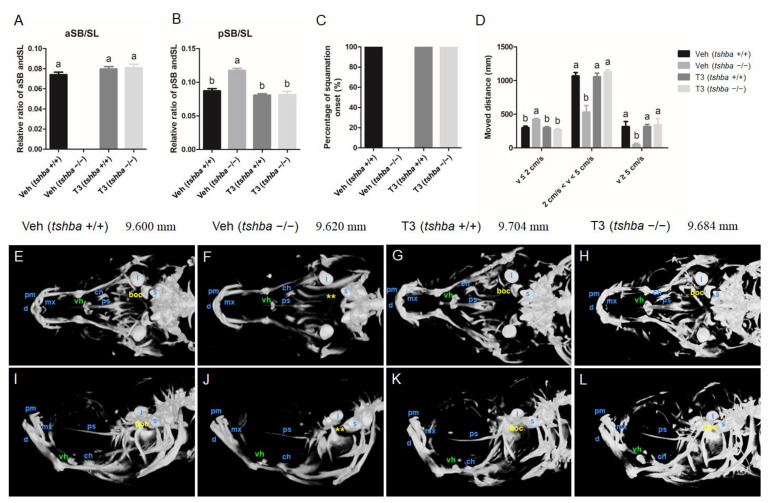
Larval-to-juvenile transition defects rescued by supplemental T3 treatments in *tshba* mutant zebrafish. (**A**) Relative ratios of the relative length of the aSB to the standard length (SL) of the zebrafish body at 20 dpf. (**B**) Relative ratios of the relative length of the posterior swim bladder (pSB) to the SL of zebrafish body at 20 dpf. (**C**) Percent onset of squamation in zebrafish at 32 dpf. (**D**) The locomotive activity of zebrafish at 30 dpf. The x-axes show the movement velocity. The swimming ability of zebrafish is shown by the moving distance with low velocity (v ≤ 2 cm/s), medium velocity (2 cm/s < v < 5 cm/s), and high velocity (v ≥ 5 cm/s). (**E**–**H**) Micro-CT scan of the craniofacial skeleton in zebrafish at 30 dpf. Dorsal view. (**I**–**L**) Micro-CT scan of the craniofacial skeleton in zebrafish at 30 dpf. Lateral view. Images not to scale. The SL of each zebrafish is presented above the panels. The basioccipital bones are labeled in yellow. The ventral hypohyals are labeled in green. Yellow asterisks indicate the bony process on the basioccipital, which did not appear in control *tshba* mutant zebrafish. Abbreviations: boc, basioccipital; d, dentary; l, lapillus; mx, maxilla; pm, premaxilla; ps, parasphenoid; vh, ventral hypohyals; s, sagitta. a–b, different superscripts in the same column indicate significant differences (*p* < 0.05).

To further correlate the defective aSB inflation and scale development with the thyroid dyshormonogenesis observed in *tshba* mutant fish, a rescue experiment with supplemental exogenous T3 treatment was performed. We treated the F2 offspring of *tshba*-heterozygote mutants with T3 dissolved in the aquarium water starting at 12 dpf. At 18 dpf, both T3- and DMSO-treated wild-type larvae developed normal inflated aSBs (Figure 4A,B).

aSB inflation was not observed in DMSO-treated *tshba* mutant larvae, and this resulted in a bigger posterior swim bladder; however, all of the T3-treated *tshba* mutant fish developed normally inflated aSBs.

At 32 dpf, scales were observed in wild-type fish but not in *tshba* mutant fish in the DMSO-treated group. However, the *tshba* mutant fish developed scales as their wild-type siblings did after T3 treatment at 32 dpf (Figure 4C). As a result, the defective phenotypes regarding aSB and SP formation during the post-embryonic development in *tshba* mutant zebrafish were effectively rescued by T3 treatment.

By 30 dpf, the swimming ability of *tshba* mutant fish was affected, compared to that of their control wild-type siblings (Figure 4D). In comparison with wild-type zebrafish, the distance that *tshba* mutant zebrafish moved with high (v ≥ 5 cm/s) and medium velocity (2 cm/s < v < 5 cm/s) decreased significantly, while the distance moved with low velocity (v ≤ 2 cm/s) increased significantly. T3 treatment rescued the locomotive defect in *tshba* mutant zebrafish. After T3 treatment (2.5 μg/L), no significant differences were observed in the distance covered at three velocity levels between *tshba* mutant zebrafish and their wild-type siblings. However, without T3 treatment, the moving activity of *tshba* mutant zebrafish was significantly different from that of the wild-type zebrafish in the control group. Thus, our results indicated that *tshba* mutant fish could have their normal swimming ability restored through external T3 supplementation.

The craniofacial skeleton of zebrafish at 30 dpf was examined under X-ray with micro-CT visualization. The cranial ossification was largely completed in wild-type zebrafish by 30 dpf (Figure 4E,I). However, general retarded craniofacial ossifications were observed in *tshba* mutant fish relative to the control wild-type fish (Figure 4F,J). The appearance of basioccipital bones was observed in wild-type fish but not in *tshba* mutant fish at 30 dpf, while the ventral hypohyal was smaller in *tshba* mutant fish compared with that of the control fish (Figure 4E,F,I,J). After T3 supplementation, the skulls were mostly ossified by 30 dpf in both wild-type and *tshba* mutant zebrafish (Figure 4G,H,K,L), and the basioccipital bones were observed in the cranial region in *tshba* mutant zebrafish.

### 3.5. Reproductive Defects Observed in Tshba Mutant Zebrafish

**Figure 5:**
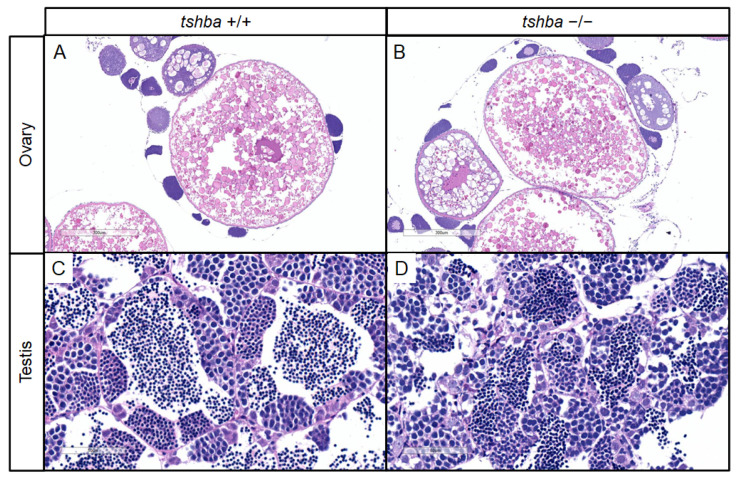

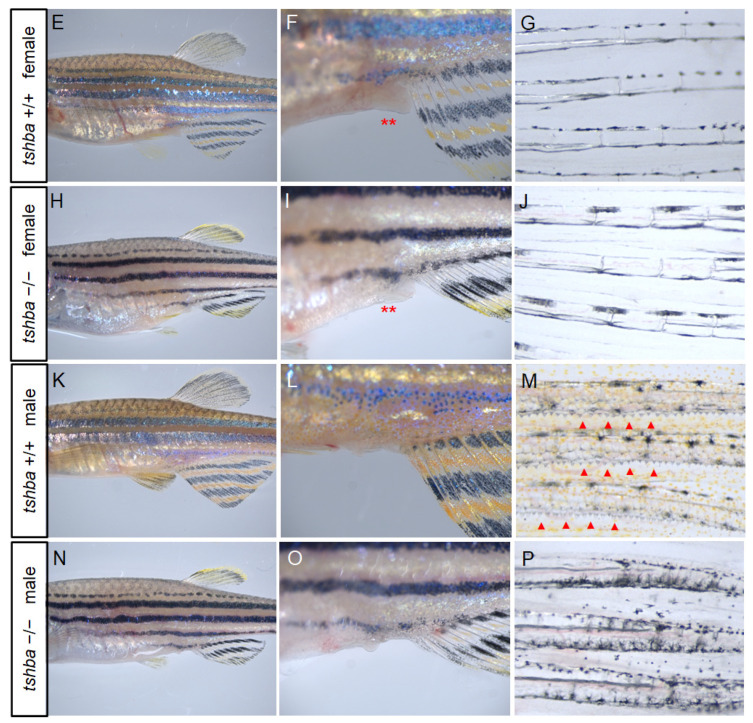
Changes in secondary sex characteristics in *tshba* mutant zebrafish. (**A**,**B**) Representative histological sections of ovaries from wild-type zebrafish at 4 mpf (**A**) and from *tshba* mutant zebrafish at 8 mpf (**B**) by H&E staining. (**C**,**D**) Representative histological sections of testes from wild-type zebrafish at 4 mpf (**C**) and from *tshba* mutant zebrafish at 8 mpf (**D**) by H&E staining. (**E**,**H**,**K**,**N**) Gross morphology of wild-type female fish at 4 mpf (**E**), *tshba* mutant female fish at 8 mpf (**H**), wild-type male fish at 4 mpf (**K**), and *tshba* mutant male fish at 8 mpf (**N**). (**F**,**I**) Representative female vent in wild-type (**F**) and *tshba* mutant (**I**) fish. Red asterisks indicate the vent anterior to the anal fin. (**L**,**O**) Representative male vent in the wild-type (**L**) and *tshba* mutant (**O**) fish. (**G**,**J**,**M**,**P**) Pectoral fins of wild-type female fish (**G**), *tshba* mutant female fish (**J**), wild-type male fish (**M**), and *tshba* mutant male fish (**P**) are shown. Representative breeding tubercles (BTs) were seen in wild-type male fish (**M**). No BTs were seen in *tshba* mutant male fish (**L**). Red arrowheads indicate the BTs on the pectoral fins.

Initially, no progeny was obtained from the mating between homozygous *tshba* mutant parents. To further address the reasons for reproductive performance defects in *tshba* mutant zebrafish, histological analyses of gonadal tissues in the *tshba* mutants were conducted. Surprisingly, the histological structures of the gonads were not affected in either male or female *tshba* mutant zebrafish, when compared with the wild-type zebrafish gonads. Similar patterns of oocytes were observed in both *tshba* mutant ovaries and those of wild-type control fish (Figure 5A,B), and similar histological features of the male germ cells were observed in both *tshba* mutant and wild-type testes (Figure 5C,D). Thus, the maturation of gonads in the *tshba* mutants was not affected.

To further address the effects of *tshba* mutation on reproduction performance, we investigated the secondary sex characteristics (SSCs) of the *tshba* mutant. A decrease in vent size was observed anterior to the anal fin in *tshba* mutant female adult zebrafish compared to that in wild-type female zebrafish (Figure 5F,I). In addition, *tshba* mutant male adult fish completely lost their breeding tubercles on the pectoral fin, while control wild-type zebrafish exhibited clear breeding tubercles (Figure 5M,P). These results demonstrate the defective development of SSCs, caused by impaired TSH signaling in zebrafish.

### 3.6. Generation of tg Mutant Zebrafish

**Figure 6:**
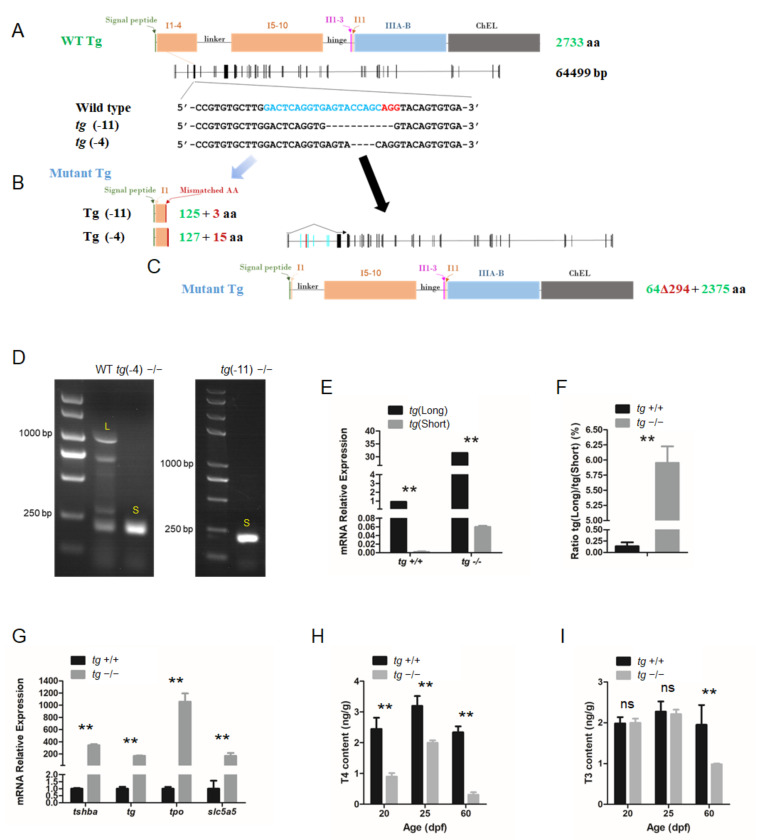
Generation of *tg* mutant zebrafish. (**A**) Schematic illustration representing the zebrafish Tg protein, the genomic structure of *tg*, and the CRISPR target site on exon 4. Sequencing of the *tg* CRISPR target site in wild type, *tg* (-4) mutant, and *tg* (-11) mutant zebrafish lines are shown in the lower panel. (**B**) The predicted truncated Tg proteins resulting from premature translational terminations are shown. (**C**) Proteins resulting from the alternative short transcript are shown. (**D**) The alternative short transcripts can be amplified in wild-type fish and *tg* mutants. The long transcripts were indicated by the letter L. The short transcripts were indicated by the letter S. (**E**) Quantification of the expression levels of the *tg* full-length transcript and short transcript in *tg* mutants and their wild-type siblings. (**F**) Ratio of the *tg* full-length transcript and short transcript expression in *tg* mutants and their wild-type siblings. (**G**) Quantitative RT-PCR analyses of *tshba*, *tg*, *tpo*, and *slc5a5* expression levels in *tg* mutants and their wild-type siblings at 5 dpf. (**H**) Whole-body T4 contents of *tg* mutants and their wild-type siblings at the 20, 25, and 60 dpf stages. (**I**) Whole-body T3 contents of *tg* mutants and their wild-type siblings at the 20, 25, and 60 dpf stages.** *p* < 0.01, ns: No significance.

The CRISPR system was used to generate *tg* mutant zebrafish. Based on the zebrafish *tg* mRNA sequence (NM_001329865.1) from GenBank, the zebrafish *tg* gene was identified as a single copy gene on Chromosome 16. The *tg* locus (LR812078.1) contains an 8202 bp coding sequence divided into 46 exons spanning over 64 kb, with 2733 predicted AAs. The targeting site was selected at the fourth exon of the *tg* gene. Two independent mutant lines, one with a 4 bp deletion (*tg*-4 line) and another with an 11 bp deletion (*tg*-11 line) were generated (Figure 6A).

Unless otherwise noted, the *tg*-4 mutant zebrafish line was used for the experiments. These mutations in the *tg* locus resulted in a reading frame shift and premature termination (Figure 6B). However, a short form of the alternative splicing *tg* transcript lacking exon 3–8 was detected in the *tg* mutant zebrafish (Figure 6C,D). Both the native full-length *tg* transcript and this alternative splicing transcript form can actually be amplified from wild-type zebrafish, but dramatically elevated levels of the short alternative transcript form were observed in the *tg* mutants (Figure 6E,F). These alternatively spliced isoforms retained many important Tg functional domains that may still perform some of the core functions of Tg. To assess the functional consequence of the *tg* mutations in zebrafish, the expression changes of genes related to thyroid function were assayed by qRT-PCR in *tg* mutant fish at 5 dpf. A general trend towards upregulation was observed, with a considerable increase of *tshba*, *tg*, *tpo*, and *slc5a*5 in the *tg* mutants (Figure 6G). Moreover, body T4 levels measured at 20, 25, and 60 dpf were significantly reduced in *tg* mutant zebrafish compared to those of wild-type zebrafish at same stages respectively (Figure 6H). T3 levels were significantly reduced at 60 dpf in *tg* mutant zebrafish, while no significant difference in T3 levels at 20 and 25 dpf was found between *tg* mutants and wild-type zebrafish (Figure 6I).

### 3.7. Generation of slc16a2 Mutant Zebrafish

**Figure 7:**
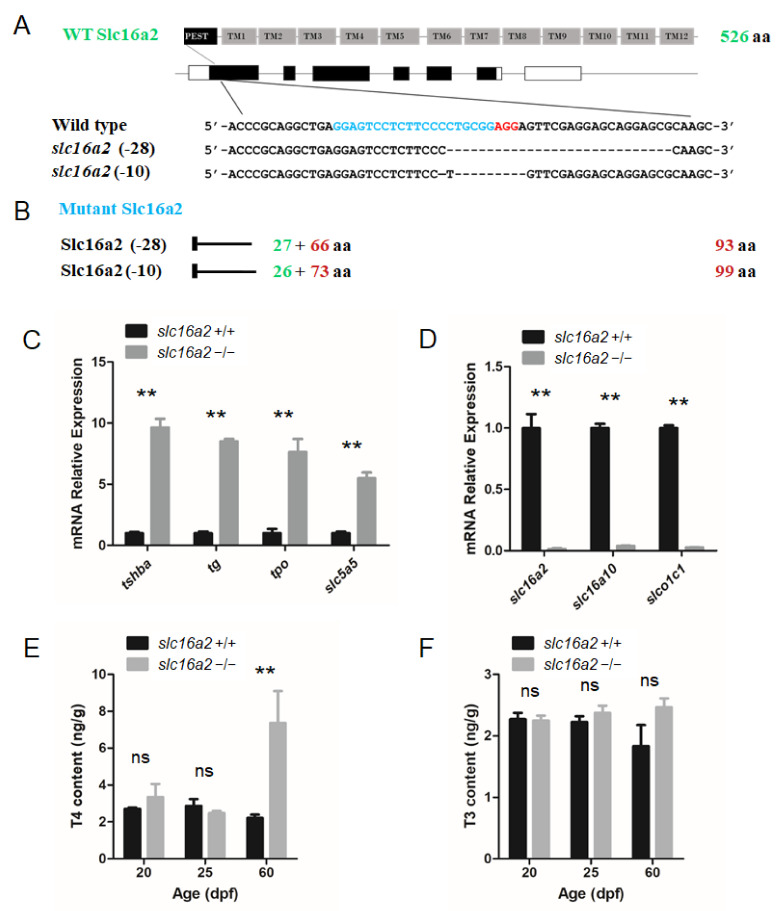
Generation of the *slc16a2* mutant zebrafish. (**A**) Schematic illustration representing the zebrafish Slc16a2 protein (upper panel) and *slc16a2* locus with the CRISPR target site on exon 1 (middle panel). Sequencing of the *slc16a2* target CRISPR site in wild type, *slc16a2* (-28) mutant, and *slc16a2* (-10) mutant zebrafish lines are shown in the lower panel. (**B**) The predicted truncated Slc16a2 proteins resulting from premature translational termination are shown. (**C**) Quantitative RT-PCR analyses of *tshba*, *tg*, *tpo*, and *slc5a5* expression in *slc16a2* mutants and their wild-type siblings at 5 dpf. (**D**) Quantitative RT-PCR analyses of *slc16a2*, slc16a10, and *slco1c1* expressions in *slc16a2* mutants and their wild-type siblings at 5 dpf. (**E**) Whole-body T4 contents of *slc16a2* mutants and their wild-type siblings at 20, 25, and 60 dpf. (**F**) Whole-body T3 contents of *slc16a2* mutants and their wild-type siblings at 20, 25, and 60 dpf. ** *p* < 0.01, ns: No significance.

The zebrafish *slc16a2* gene (NW_003335266.1) was also targeted using the CRISPR system. The targeting site was chosen in the first exon of the *slc16a2* gene. Two independent mutant lines, one with a 10 bp deletion (*slc16a2–*10 line) and another with a 28 bp deletion (*slc16a2–*28 line) were generated (Figure 7A).

Unless otherwise noted, the *slc16a2–*28 mutant zebrafish line was used for experiments. The mutations in *slc16a2* resulted in the reading frame shift and premature terminations (Figure 7B). In these mutant fish at the 5 dpf stage, the expression levels of *tshba*, *tg*, *tpo*, and *slc5a5* were significantly upregulated in *slc16a2* mutant zebrafish (Figure 7C). One remarkable exception was the dramatic reduction in three thyroid hormone transporters (*slc16a2*, *slc16a10*, and *slco1c1*) in *slc16a2* mutant zebrafish (Figure 7D). In terms of the TH production in *slc16a2* mutant fish, the T4 levels of *slc16a2* mutant zebrafish significantly increased at 60 dpf compared to those of wild-type zebrafish, while the T4 levels at 20 and 25 dpf were not significantly affected (Figure 7E). In terms of T3 production, no significant change was observed at 20, 25, and 60 dpf in *slc16a2* mutant zebrafish (Figure 7F).

### 3.8. General Phenotype Observed in tg and slc16a2 Mutant Zebrafish

**Figure 8:**
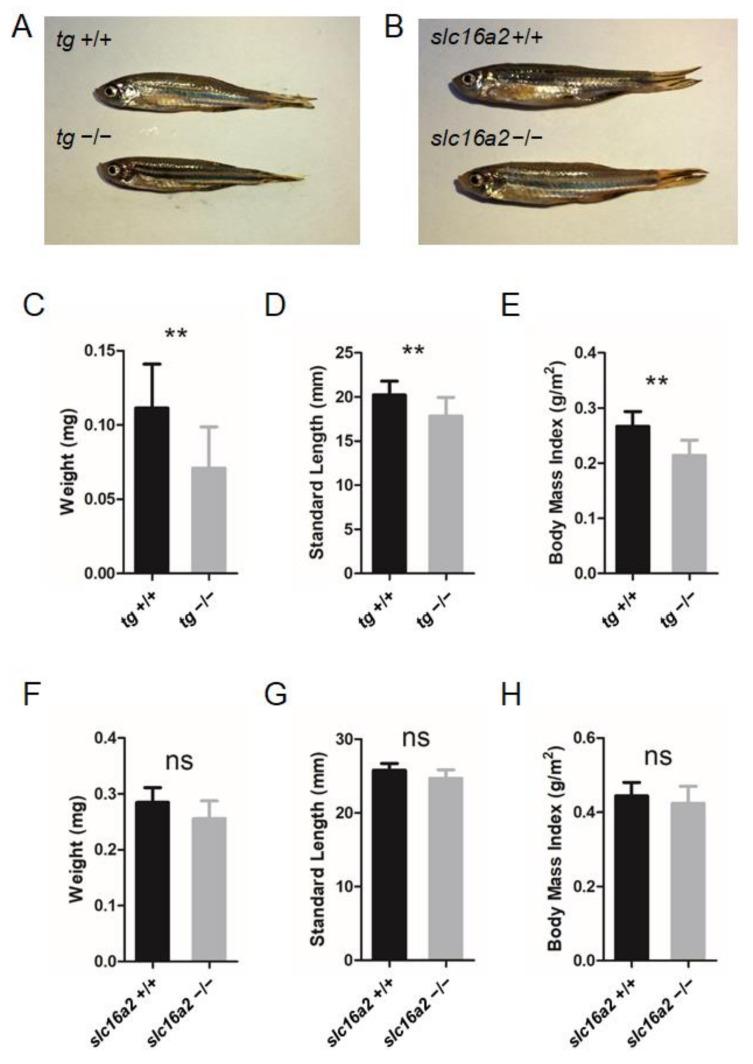
Somatic growth in *tg* or *slc16a2* mutant zebrafish. (**A**) Control wild-type zebrafish and *tg* mutant zebrafish at 2 mpf. (**B**) Control wild-type zebrafish and *slc16a2* mutant zebrafish at 2 mpf. (**C**–**E**) Body weight (**C**), standard length (SL) (**D**), and body mass index (BMI) (**F**) of *tg* mutants and their wild-type siblings at 2 mpf. Measurements were taken from wild-type fish (n = 12) and *tg* mutant fish (n = 13). (**F**–**H**) Body weight (**F**), SL (**G**), and BMI (**H**) of *slc16a2* mutants and their wild-type siblings at 2 mpf. Measurements were taken from wild-type fish (n = 6) and *slc16a2* mutant fish (n = 8); ** *p* < 0.01, ns: No significance.

Since significant alterations in certain TH levels of *tg* and *slc16a2* mutant zebrafish were observed at 60 dpf (Figure 6H,I and Figure 7E,F), the morphological features of the mutant fish were subsequently investigated. Both *tg* and *slc16a2* mutant zebrafish exhibited normal shapes and pigmentation patterns as those of wild-type zebrafish (Figure 8A,B).

**Figure 9:**
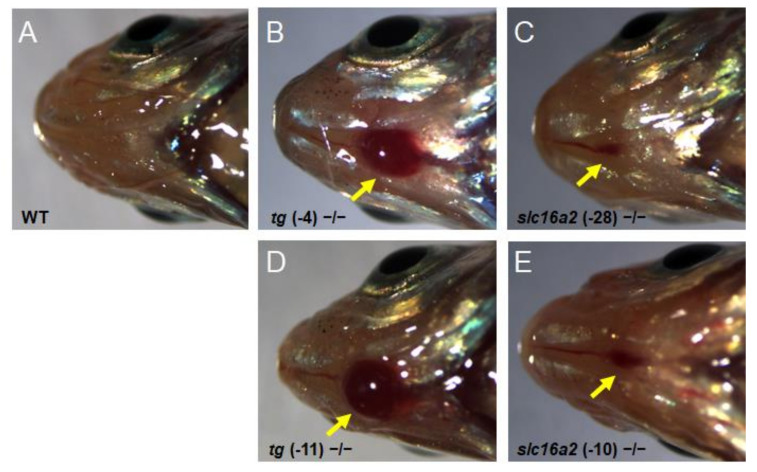
Goiter seen in *tg* mutant and *slc16a2* mutant zebrafish. (**A**–**E**) Representative macroscopical pictures of the head region of wild-type zebrafish (**A**), *tg* (-4) zebrafish (**B**), *tg* (-11) mutant zebrafish (**C**), *slc16a2* (-10) mutant zebrafish (**D**), and *slc16a2* (-28) mutant zebrafish (**E**) at 3 mpf. The red mass of the thyroid is indicated by the yellow arrows.

Bodyweight and SL of zebrafish at 60 dpf were measured. The weight and length of the *tg* mutant fish were both significantly lower than those of wild-type fish; correspondingly, the BMI was reduced in *tg* mutant zebrafish (Figure 8C–E). Unlike the *tg* mutants, the body weight, body length, and BMI of *slc16a2* mutant fish were not significantly affected compared to those of their wild-type siblings (Figure 8F–H). Notably, no apparent goiter was found in *tshba* mutant zebrafish (Supplementary Figure S2), while typical goiters of a large red protrusive mass were observed under the jaw and anterior to the heart in *tg* and *slc16a2* mutant zebrafish at 3 mpf (Figure 9A–E), and the mass in *tg* mutant zebrafish was larger than that in *slc16a2* mutant zebrafish. These results suggest that mutations of *tg* and *slc16a2* cause goiter in zebrafish.

Due to defects of inflation of aSB and the onset of scales in *tshba* mutant fish, we subsequently investigated the development of aSB and scales in *tg* and *slc16a2* mutant fish. At 20 and 32 dpf, no evident defects of the key features involved in LTJT were observed in either *tg* or *slc16a2* mutant fish (Supplementary Figure S3). These results indicated that the LTJT was not affected in either *tg* or *slc16a2* mutant zebrafish.

### 3.9. Divergent Effects of Reproduction in Various Zebrafish Hypothyroidism Models

**Figure 10:**
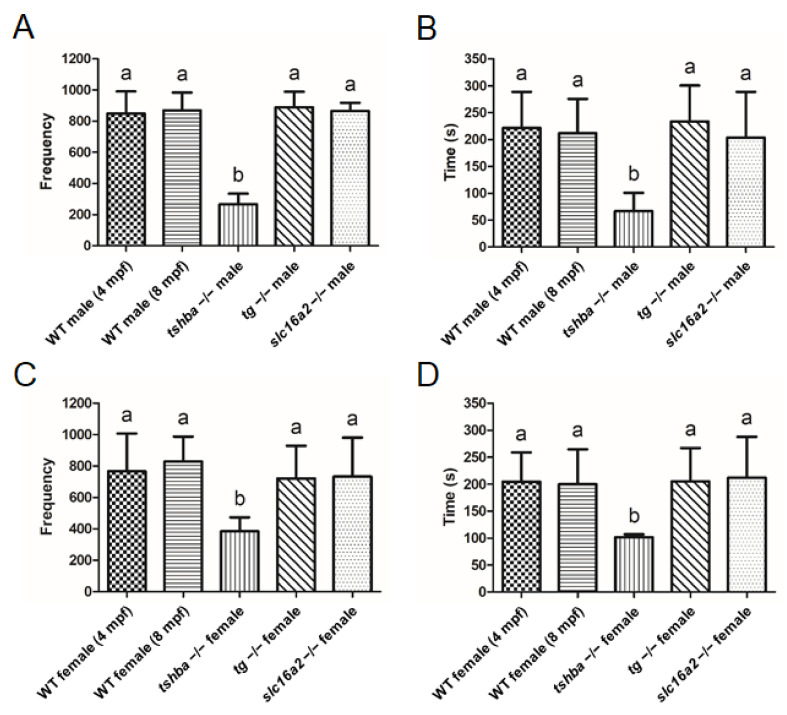
Reproductive behavior analyses in wild-type control fish and *tshba*, *tg*, and *slc16a2* mutant zebrafish. (**A**,**B**) Mating behavior exhibited by wild-type female zebrafish towards the mutant male fish. Frequency (**A**) and duration (**B**) of intimate contacts are shown. (**C**,**D**) Mating behavior exhibited by wild-type male zebrafish toward the mutant female fish. Frequency (**C**) and duration (**D**) of intimate contacts are shown. Measurements were taken from wild-type control males (4 and 8 mpf) and females (4 and 8 mpf), *tg* mutant males (4 mpf) and females (4 mpf), *slc16a2* mutant males (4 mpf) and females (4 mpf), and *tshba* mutant males (8 mpf) and females (8 mpf). n = 3 per group. a–b, different superscripts in the same column indicate significant differences (*p* < 0.05).

No evident defective reproductive activities were identified in homozygous *tg* or *slc16a2* mutants, while no progenies were produced from *tshba* mutants. To further analyze the effects of various hypothyroidisms on reproductive performance, the mating behaviors of wild-type fish (4 and 8 mpf), *tshba* mutants (8 mpf), *tg* mutants (4 mpf), and *slc16a2* mutants (4 mpf) were examined. No significant differences were found between the frequency and duration of intimate contact (<2 cm) among wild-type fish at 4 mpf and wild-type fish at 8 mpf in both sexes (Figure 10A–D).

There was significantly reduced frequency and duration of intimate contact when mating was set up between *tshba* mutant males with wild-type females (4 mpf). However, no such phenomenon was observed when the mating involved *tg* or *slc16a2* mutant male zebrafish (Figure 10A,B). Consistently, the *tshba* mutant female fish exhibited significantly decreased intimacy with wild-type male fish (4 mpf), while the mating behavior of *tg* and *slc16a2* mutant female zebrafish with wild-type males (4 mpf) was not significantly affected (Figure 10C,D). Hypotestosterone levels may be caused by a specific form of hypothyroidism in mammals [40]. As the testosterone level was generally considered to be associated with reproductive activities, the testosterone levels in the gonads of wild-type fish (4 and 8 mpf), *tshba* mutants (8 mpf), *tg* mutants (4 mpf), and *slc16a2* mutants (4 mpf) were measured. The gonadal testosterone levels among wild-type fish at 4 mpf and wild-type fish at 8 mpf showed no significant differences in either sex. Moreover, no significant changes in testosterone levels of the testes or ovaries were found in *tshba*, *tg,* or *slc16a2* mutant zebrafish compared to those of the control wild-type fish (4 mpf) (Supplementary Figure S4). Therefore, this indicates that the reproductive defects observed in *tshba* mutants are not associated with the gonadal levels of testosterone.

## 4. Discussion

The life cycle of teleost fish and anuran amphibians can be divided into the following five stages: zygote, embryo, larva, juvenile, and adult [41]. During amphibian metamorphosis, many TH-dependent morphological changes occur and require a gradual increase in plasma TH levels from very low in pre-metamorphic tadpoles to peak levels at the climax stages. Towards the end of metamorphosis, TH plasma levels decrease [41,42]. This amphibian metamorphosis is generally most prevalent during LTJT [43]. Many teleosts do not show dramatic changes in morphology from the larval to the juvenile stage. However, TH signaling may be necessary for the proper developmental remodeling process in zebrafish. Originally, the role of THs in zebrafish development was studied using goitrogen treatments [44,45,46], which biologically block TH synthesis in thyrocytes and induce the hypothyroid state. The conclusions of the LTJT studies with goitrogen treatments can be confusing as the chemicals used in these studies also generate risks due to toxicity associated with non-TH-related side effects [47]. Regarding the functions of endogenous TH signaling, some studies have been conducted that introduce antisense morpholino oligonucleotides (MOs) targeting certain TH-related molecules [48,49,50,51]. The phenotype with decreased body length and weight, delayed swim bladder inflation, and reduced motility has been analyzed [47]. However, gene silencing via MO experiments limits the elucidation of the functional studies to stages prior to 6 dpf in zebrafish development, which is not extended to the LTJT period. The T3:T4 ratio, which represents monodeiodinase activity, peaks at 14 dpf in zebrafish and may be critical for LTJT [41]. Recently, some genes, such as TH transporters (Slc16a2) and DIOs D1, D2, and D3, were targeted to generate zebrafish hypothyroidism models for peripheral TH functional studies. Defective locomotor activities have been observed in both *slc16a2* and *dio2* mutant zebrafish, while the defects in swim bladder inflation and fertility have only been observed in *dio2* mutant fish [24,52]. Considering the multi-analogous members and unique tissue distribution patterns of each TH transporter and DIO, the defective phenotypes observed with these models mainly involved tissue-specific or local effects [52,53]. In humans, the primary sources of reactive oxygen species production for TH synthesis are NADPH oxidases. Two NADPH oxidase genes, *duox* and *duoxa2*, have been identified in zebrafish [54]. In 2019, Chopra et al. generated *duox* mutant zebrafish, which could be a potential CCH model. Unfortunately, the authors did not measure the TH levels in the *duox* mutant fish, but noted that no detectable T4 signaling occurred in the thyroid region of the *duox* mutants at 5 dpf, identified via wholemount fluorescent immunohistochemistry. However, some phenotypes related to TH signaling, such as ragged fins, goiter, and infertility, have been characterized in *duox* mutant fish and heart defects in *thrα* mutant fish. These prior studies did not focus on the LTJT period [26,27]. Generally, phenotypes, such as growth retardation and pigmentation defects, are commonly seen in many mutant zebrafish with deficiencies in TH signaling-related molecules [26,52]. Some phenotypes involved in the LTJT process, such as aSB inflation, jaw morphology, feeding behaviors, cranial ossification, and scale formation, are related to TH signaling [26,52,53,55]. However, to our knowledge, no previous studies have characterized all of these features during a suggested LTJT period based on systematic observations of a single CCH model.

The TSH signal plays key functions in the regulation of endogenous TH homeostasis in animals. However, TSH receptor (*tshr*) null mice have been generated that have severe defects and die within one week of weaning [56], making this model unsuitable for exploring the function of TSH signaling on LTJT. Surprisingly, no *tshb* null animal models, except for some identified natural *tshb* mutations from patients, have been reported previously. The mutation C105V in human *tshb*, the most common mutation that causes TSH deficiency, was first described in 1996, which causes severe signs of CCH, including hypothermia, lethargy, muscle hypotonia, prolonged jaundice, and delayed closure fontanelles [57]. This TSHb mutation results from the last cysteine being replaced, which can modify the TSHb structure and deteriorate the heterodimeric TSH complex, which potentially leads to the inactivation of TSH or to its interplay with the TSHR. Patients with such a TSHb gene mutation show a severe phenotype compared to those with athyreosis [9]. Based on the identified fish *tshb* genes, most of the teleost TSHβ proteins ranged in size from 139 to 165 AAs, with 12 conserved cysteine residues and a functional domain region and exhibited strong similarities in terms of sequences with their mammalian counterparts (Supplementary Figure S1A). Like mammalian TSHβ protein, six conserved cysteine pairs in fish TSHβ protein can form the specific structure of the glycoprotein hormone, including the seat-belt structure, which wraps around the GSUα protein domain to form a stable heterodimer structure, thereby forming the TSH complex [58]. In the present study, a *tshba*-mutant zebrafish line was generated. A 5 bp deletion in the third exon of the zebrafish *tshba* locus resulted in a premature stop codon in the putative mutant Tshba protein. This protein may deteriorate the formation of the stable heterodimer TSH protein due to the absence of the last Cys of the 12 conserved Cys sites and a damaged seat-belt structure. Compared with their wild-type control siblings, elevated *tshba* mRNA (mutant form) but not *tshbb* mRNA in our homozygous *tshba* mutant was observed. Besides, the reduced transcriptional levels of *tg*, *tpo*, and *slc5a5* and protein levels of TG have been recorded in *tshba* mutants compared with those of the control fish. The results of this study indicate that thyroid dyshormonogenesis is caused by the Tshba mutation and the effects of compensation of the impaired TH production via Tshba in the *tshba* mutant, but not via the *tshbb* in zebrafish. Since both adult males and females of homozygous *tshba* mutant fish were infertile, all fish used in our study were obtained from the mating of heterozygous *tshba* mutant parents. Thus, the effects of the maternal TSH in *tshba* mutant embryos cannot be excluded. Consistent with the results of previous studies regarding zebrafish thyroid development [59], *tshba* was first expressed between 28 and 32 hpf in the pituitary [60]. The co-expression of *tshb* and *gsu* in mutants was detected at 48 hpf, indicating the initiation of differentiated thyrotropes. No significant difference in TH levels between *tshba* mutants and wild-type controls was observed prior to the 20 dpf stage, although there was a significant reduction in TG levels in the *tshba* mutant at 5 dpf. This suggests that TH synthesis may not be dependent on the autonomous synthesis of TSH during the early developmental stages. The significantly decreased TH levels were only detected up until 20 (T4) and 25 (T3) dpf in *tshba* mutant fish (Figure 1L,M), which coincides with the critical period for LTJT. In mice, TSHR may exhibit the constitutive basal signaling activity responsible for a minimal level of TH production [9]. If this is the case in zebrafish, it is possible that the basal signaling responsible for minimal TH production and active thyroid hormonogenesis, which are needed to promote critical developmental remodeling, such as LTJT, escalate endogenous TH production via TSH signaling.

In the state of hypothyroidism, TSHR null mice were severely runted and wasted during the pre-weaning period and died within one-week post-weaning [56]. Growth retardation was also exhibited in *duox* null zebrafish at three months of age [26]. The somatic growth retardation of our *tshba* mutant fish could only be distinguished from their wild-type control siblings after 35 dpf (Figure 2D). Thus, all morphological and behavioral features of the *tshba* mutant fish were investigated thoroughly during the post-embryonic stages prior to 35 dpf. Previous studies based on easily identified external morphological traits (e.g., pigment pattern and fin morphology) have suggested that the majority of zebrafish reach the larval and juvenile stage at 5- and 12-mm SL [39,55]. In our study, the first altered signs detected were the delay of aSB inflation in Tshba mutant fish at their 6 mm SL stage. This stage occurred around 16 dpf under our culture conditions. Significant differences in somatic growth and SL were seen after the 12 mm SL stage and 35 dpf in both mutant and wild-type zebrafish. On the other hand, the period of active thyroid hormonogenesis in zebrafish coincided with the time when the significant alteration between the TH production of *tshba* mutant and wild-type fish was observed at 16–35 dpf (Figure 1L,M). Therefore, 6–12 mm SL and 16–35 dpf should be considered as the LTJT period for zebrafish. Previously, the initial defects in pigment patterns and aSB in Tg (tg: nVenus-2a-nfnB) zebrafish models were recorded at the 10 mm SL and 5.5–8.5 mm SL stages, respectively [61]. In a *duox*-deficient zebrafish hypothyroidism model, aSB defects were observed at 21 dpf [26]. This timing is in accordance with the results of the present study in terms of the suggested zebrafish LTJT period. Overall, beginning at the larval stage (6.1 mm SL), we detected the phenotypes of the defective aSB inflation, SP appearance, metamorphic melanophore pattern, pelvic fin fold absorption, and cranial skeleton development in our *tshba* mutant fish. Reduced moving activity of the *tshba* mutant fish at 30 dpf was also recorded (Figure 4D). The observed impaired features have been detected in many phenotypes of *dio2*-deficient zebrafish and *slc16a2*-morphants [52,62]. Along with the significantly reduced TH levels observed in the LTJT period of the *tshba* mutant fish, these results demonstrated that functional TSH signaling might be critical for stimulating active TH production to promote the onset of developmental remodeling of young zebrafish and account for fitness-related parameters during LTJT.

Juvenile fish must undergo physiological rearrangements to cope with their free-swimming life during LTJT, even in a direct-developing teleost zebrafish model. TH signaling in zebrafish may play a major role in regulating aSB inflation, jaw morphology, feeding behaviors, and cranial ossification [42,52,63,64]. However, there have been no systemic studies, to our knowledge, that focus on the array of developmental remodeling that occurs during LTJT using a CCH model. In the present study, we demonstrated that *tshba* mutant zebrafish as a CCH model due to the impaired TH production dynamics display defects, including aSB inflation and cranial ossification (Figure 1L,M, Figure 3C and Figure 4E–L). At the end of the LTJT period, the *tshba* mutant fish exhibited movement defects (Figure 4D). Appropriate dynamic TH production is required for the development of zebrafish feeding mechanics via cranial ossification, which may be critical to promote their diversification into adult feeding niches [63]. In cyprinids, aSB may play an important function in hearing through its connection with the ear via the Weberian apparatus [65]. Analyses using our hypothyroidism model demonstrated differential TH dependencies of LTJT events in zebrafish via the TSH signal, and their growth retardation could be a consequence of the defects observed during LTJT (Figure 2D–G). This study established a classic CCH zebrafish model, which may be useful for studying the etiology and underlying mechanism of some human hypothyroidism diseases.

Zebrafish TG is a large, secreted glycoprotein with a predicted 2734 AA that comprises a signal peptide and I1–4 and I5–10 regions at its N terminal. Previous studies with mammalian models have indicated that region I is critical for the TG maturation process and functions involved in the regulation of cysteine or cation-dependent protease activity, as well as long-term iodide storage for iodotyrosine coupling for hormonogenesis [66]. In mammals with a large number of exons and various exonic sizes separated by introns spanning a long range in genomic DNA, Tg transcripts are very heterogeneous due to the polymorphisms. Constitutive and alternative splicing in the human *tg* gene is complex [67]. In the present study, besides the *tg* transcript (long form), which is similar to the full-length *tg* mRNA deposited in GenBank, a very minor fraction of the total *tg* transcripts was detected as the alternative splicing form of the *tg* transcript (short form) in wild-type zebrafish (Figure 6E,F). The short alternative splicing *tg* transcript form skips five complete exons (exons 3–8) with the remaining transcript in the same frame as the full-length *tg* mRNA, suggesting a short form of TG protein with the I1–4 (293 AAs) region missing. In *tg* mutant fish, the transcriptional level of the short form alternative transcript increased significantly, especially compared with the level of full-length *tg* transcript containing the 4 bp missing indel (Figure 6D–F). This might be due to the compensation effects of the premature termination of the long form *tg* transcript in the -4 or -11 *tg* mutant mRNAs in these *tg* mutant fish, since the putative peptides of the -4 or -11 *tg* transcript could only be translated to a 128 AA peptide, missing almost all the functional domains in native TG protein. In humans, an estimated thyroid dyshormonogenesis incidence of approximately 1/100,000 newborns has been reported due to more than 100 deleterious mutations in the *TG* gene [14]. The typical phenotypes of thyroid dyshormonogenesis were found in our *tg* mutant zebrafish, with significantly decreased levels of T4 and T3 production (Figure 6H–I) as well as growth retardation (Figure 8A,C–E), suggesting the poor functioning of the short TG form encoded by the *tg* alternative splicing form without the I1–4 region.

Unlike the central functions of TSH and TG in the HPT axis, the SLC16A2 as a TH membrane transporter plays a local TH regulatory role in peripheral organs [47]. Therefore, there were much less severe defects in our *slc16a2* mutant fish, compared with those observed in the *tshba* and *tg* mutants. No significantly altered levels of T3 or growth retardation were recorded in the *slc16a2* mutants (Figure 7F and Figure 8B,F–H). *slc16a2* mutant zebrafish have also previously been generated via the CRISPR/Cas9 technique, with the targeting site at a similar region to that of the *slc16a2* mutant zebrafish targeted via the ZFN technique previously [24]. However, significantly upregulated levels of *tshba* were found in our *slc16a2* mutants at 5 dpf, while no significant alteration of *tsh* expression was found in the *slc16a2* mutant at 3 dpf described previously. Significantly increased levels of *tg* expression and T4 at 60 dpf were found in the *slc16a2* mutants, while no information has previously been provided, to our knowledge, regarding this mutant [24].

One in eight women in the USA will develop a thyroid goiter at some point in their lifetime as a result of various factors [68]. Only a few of these incidences will be congenital goiters. Normally, the goiters can be classified as hypothyroid, euthyroid, or hyperthyroid [11,69]. In all cases, the roles of TSH are critical. Normally, thyroid dyshormonogenesis induces TSH production and, as a consequence, a goiter phenotype occurs in various hypothyroidism models [11]. In patients with a euthyroid goiter, the HPT axis plays a role within a relatively broad range of TSH levels to keep the TH production on track via the regulation of the thyroid gland function. Graves’ disease is the most common cause of hyperthyroidism, resulting from endogenous autoantibodies in the TSHR that overactivate the receptor, thereby stimulating TH synthesis and secretion as well as a diffuse goiter [70]. In the mutant zebrafish models used in this study, euthyroid goiters were observed in *slc16a2* mutants, with relatively normal levels of T3 without other evident phenotypes (Figure 7F, Figure 8F,G and Figure 9C,E). A typical phenomenon of hypothyroid goiter was found in the *tg* mutant fish, with evident defects of TH production and growth (Figure 6H,I, Figure 8A,C–E and Figure 9B,D) similar to the defects seen in *duox* mutant zebrafish [26]. Significantly elevated *tshba* expression in both *tg* and *slc16a2* mutants indicated the role of TSH in goiter formation. No goiter was observed in the CCH model, which further demonstrated both the essential function of TSH on the promotion of goiter and the deteriorated function of the mutated Tshba protein in the autonomous regulation of thyroid hormonogenesis in zebrafish.

Previously, reproductive dysfunction with relatively normal gamete development was observed in two hypothyroidism models: *duox* and *dio2* zebrafish mutants [26,71]. More studies regarding *dio2* mutant fish also indicated that the sex steroid production was downregulated by *dio2* deficiency [71]. However, reproductive activities in the *tg* mutants are normal, although this seems like a typical hypothyroidism model in zebrafish. However, similar observations regarding gamete development and mating behavior in the *tshba* mutants, another CCH model, were found (Figure 5A–D and Figure 10A–D). Impaired development of the SSCs was found in both genders of *tshba* mutant fish (Figure 5E–P). In our previous study with *cyp17a1*-deficient zebrafish, it was demonstrated that the levels of testosterone were critical to SSC development [72]. However, no significant alterations of testosterone levels in adult *tshba*, *tg*, and *slc16a2 mutants* have been found (Supplementary Figure S4), which is different from the observations of the adult *dio2*-deficient zebrafish [71]. Based on the observations from our two CCH models, the *tshba* mutants exhibit defective LTJT features, such as a delay in the inflation of the aSB, but the *tg* mutants do not. By comparing the dynamic TH production patterns between *tshba* and *tg* mutants, it was found that an earlier significant decrease in T3 levels occurred in the *tshba* mutant compared to the *tg* mutant. This might suggest that the early alteration of T3 levels is critical for the proper development of mating behavior, and this behavior is not related with the decreased levels of steroid hormone in hyperthyroidism zebrafish models (Supplementary Figure S4). Although no information regarding the beginning of the significantly decreased TH levels in *duox* and *dio2* mutants is available from previous studies [26,71], it is noteworthy that both defects of mating behavior and inflation of aSB occurred in *duox*, *dio2*, and *tshba* mutants (Figure 3, Figure 4, Figure 5 and Figure 10) [26,71], but neither defect were seen in *tg* mutants (Figure 10 and Figure S3). This might suggest the possible connection between the LTJT and SSC development in zebrafish.

Teleosts undergo certain post-embryonic metamorphic transitions. The present study demonstrated systematic analyses on the justification of the LTJT period and the endocrine regulation of the major features that occur during the key developmental remodeling processes of LTJT in zebrafish, with the *tshba* mutant model. Our three thyroid disorder models also provided various goiter models, which further justify the essential roles of TSH involved in zebrafish goiter development. Intriguingly, on combining our observations on *tshba*, *tg*, and *slc16a2* mutants and previous reports on two other hyperthyroidism zebrafish models, *duox* and *dio2*, the involvement of TH signaling in both LTJT, and SSC development can be found. This study verifies the critical function of TSH signaling on promoting LTJT in zebrafish, which is analogous to the metamorphosis of anurans and provides important insights into the evolution of animal LTJT (or metamorphosis) and the identification of the key diversifications of physiology, behavior, and morphological features, and possibly includes SSC formation during the teleost LTJT period. More subsequent investigations are needed for the regulatory mechanisms of TH signaling in the somatic growth and SSC formation of our hyperthyroidism teleost models.

## Data Availability

Not applicable.

## Supplementary Materials

The following are available online at https://www.mdpi.com/article/10.3390/cells10081984/s1, Figure S1. Comparison of the protein sequence TSHb subunits, Figure S2. Histological features of the thyroid gland in *tshba* mutant zebrafish, Figure S3. No evident defects during larval-to-juvenile transition were observed in *tg* or *slc16a2* mutant zebrafish, Figure S4. No evident changes in testosterone content in the gonads of all three mutant fish types, Table S1. Primers used for PCR reaction, Table S2. The primers used for the amplification of *tg* alternative short transcripts.
