# Supplementary material for: Functions of the Thyroid-Stimulating Hormone on Key Developmental Features Revealed in a Series of Zebrafish Dyshormonogenesis Models
Source: Cells. 2021 Aug 4;10(8):1984. doi: 10.3390/cells10081984 (PMC8391828; doi:10.3390/cells10081984)
Supplement: Supplementary file 1 [file cells-10-01984-s001.zip › cells-1269928-supplementary.pdf]

Supplementary Materials

Functions of the thyroid-stimulating hormone on key developmental features revealed in a series of zebrafish dyshormonogenesis models

Jia Song [1,2], Yao Lu [1,2], Xiaoxia Cheng [3], Chuang Shi [1], Qiyong Lou [1], Xia Jin [1], Jiangyan He [1], Gang Zhai [1,2,*], Zhan Yin [1,2,*]

[1] State Key Laboratory of Freshwater Ecology and Biotechnology, Institute of Hydrobiology, Chinese Academy of Sciences, Wuhan 430072, People's Republic of China

[2] College of Advanced Agricultural Sciences, University of Chinese Academy of Sciences, Beijing 100049, People's Republic of China

[3] Key Laboratory of Receptors-Mediated Gene Regulation and Drug Discovery, School of Basic Medical Sciences, Henan University, Kaifeng 475004, People's Republic of China

*** Corresponding author:** Zhan Yin and Gang Zhai, State Key Laboratory of Freshwater Ecology and Biotechnology, Institute of Hydrobiology, Chinese Academy of Sciences, Wuhan 430072, People's Republic of China

Running title: Thyroid-stimulating hormone on zebrafish development

Key words: thyroid-stimulating hormone, thyroglobulin, *slc16a2*, larval-to-juvenile transition, goiter, secondary sex characteristic

Supplementary figure legends

Figure S1. Comparison of the protein sequence TSHb subunits. (A) Amino acid (AA) sequence alignment and phylogenetic tree of TSHb subunit AA sequences. The analyses were carried out with the CLUSTALW program online. The accession numbers for the proteins are as follows: human TSHb (NP_000540.2), mouse TSHb (NP_001159411.1), rainbow trout TSHb (NP_001118015.1), zebrafish TSHba (AAI63604.1), and zebrafish TSHbb (XP_017209108.2). The signal peptide is indicated by the green box. Glycosylation sites are shown in blue. Glycosylation sites are highlighted in blue. Cysteine residues for intramolecules are highlighted in green. Overall conserved AAs are indicated in red. Cysteine knots for the dimer interface are labeled with an asterisk below the sequence. Additional sequences, the miscoded peptides of human TSHb C105V mutant, and zebrafish TSHba mutant are highlighted in brown. (B) Inset: the phylogenetic analyses of the TSHb protein sequences.

Figure S2. Histological features of the thyroid gland in *tshba* mutant zebrafish. (A-B) Representative macroscopical pictures of the head region of wild-type zebrafish (A) and *tshba* mutant zebrafish (B) at 2 mpf. (C-D) Histopathology of thyroid follicles from wild-type fish (C) and *tshba* mutant fish at 2 mpf. The isolated thyroid follicles are distributed adjacent to the ventral aorta in the gill region. (E-F) High magnification views of thyroid follicles seen in wild-type control fish (E) and *tshba* mutants (F) at 2 mpf. Thyroid follicles are labeled with yellow asterisks. The follicular shape is spherical-to-oval with a cuboidal epithelium and a homogeneously stained colloid. Follicles from

the *tshba* mutant zebrafish with a homogeneous colloid but lower colloidal density. Sections of 2 µm thickness stained with periodic acid-Schiff and Mayer's hematoxylin.

Figure S3. No evident defects during larval-to-juvenile transition were observed in *tg* or *slc16a2* mutant zebrafish. (A) Standard length (SL) of *tg* mutants and their wild-type siblings at 20 dpf. (B) Relative ratios of the relative length of the aSB to the SL in *tg* mutants and their wild-type siblings at 20 dpf. (C) Relative ratios of the relative length of the pSB to the SL in *tg* mutants and their wild-type siblings at 20 dpf. (D) SL of the *slc16a2* mutant and their wild-type siblings at 20 dpf. (E) Relative ratios of the relative length of the aSB to the SL in *slc16a2* mutants and their wild-type siblings at 20 dpf. (F) Relative ratios of the relative length of the pSB to the SL in *slc16a2* mutants and their wild-type siblings at 20 dpf. (G) Percent onset of squamation in the *tg* mutants, *slc16a2* mutants, and their wild-type siblings at 32 dpf.

Figure S4. No evident changes in testosterone content in the gonads of all three mutant fish types. (A) Testosterone contents in the testicular tissue from adult male fish. (B) Testosterone contents in the ovarian tissue from adult female fish. Measurements were taken of wild-type control males (4 and 8 mpf) and females (4 and 8 mpf), *tg* mutant males (4 mpf) and females (4 mpf), *slc16a2* mutant males (4 mpf) and females (4 mpf), and *tshba* mutant males (8 mpf) and females (8 mpf) for analyses. n = 3 per group.

Supplementary figures

Figure S1

A

```
Zebrafish TSHbb        MKRCCHWCCCHVEFSLEDQHEDTPPQKLHKSRRAAQIRLQTTSRQTLHTGPLHCRYKLFV    60
Human TSHb             -----------------------------------------------------------M    1
Mouse TSHb             -----------------------------------------------------------M    1
Rainbow TSHb           -----------------------------------------------------------M    1
Zebrafish TSHba        ------------------------------------------------------------    0

Zebrafish TSHbb        RMRVLLCSFLLLLGEDALLACSLKNYTLYVEKHECGHCMAINTTVCSGMCFTRDTNVQGF    120
Human TSHb             TALFLMSMLFGLTCGQAMSFCIPTEYTMHIERRECAYCLTINTTICAGYCMTRDINGKLF    61
Mouse TSHb             SAAVLLSVLFALACGQAASFCIPTEYTMYVDRRECAYCLTINTTICAGYCMTRDINGKLF    61
Rainbow TSHb           ELSVAMYGLLCLLFSQAVPMCVPTDYTLYEERRECDFCVAINTTICMGFCYSRDSNMKEL    61
Zebrafish TSHba        MSLLYVIGMLGLLMKVAVPMCAPTDYTIYIERQECNYCVAVNTTICMGFCFSRDSNIKEL    60
                                          *         *      #   @      ************

Zebrafish TSHbb        VGKRFLLQQSCMHRSLVYRSARMPGCPVHIDPLFFYPVARRCNCTKCNTSRNECVFRHK-    179
Human TSHb             LPKYALSQDVCTYRDFIYRTVEIPGCPLHVAPYFSYPVALSCKCGKCNTDYSDCIHEAIK    121
Mouse TSHb             LPKYALSQDVCTYRDFIYRTVEIPGCPHHVTPYFSFPVAISCKCGKCNTDNSDCIHEAVR    121
Rainbow TSHb           AGPRFLIQRGCTYDQVEYRTVILPGCPLHANPLFTYPVALSCHCGTCNTDSDECAHKASS    121
Zebrafish TSHba        VGPRFIVQRGCTYQEVEYRTAVLPGCPSHADPHFTYPVALSCHCSTCKTHSDECALKTR-    119
                                  **                                  * *   ******

Zebrafish TSHbb        HKHNRCSKQLRTV-----------------    192
Human TSHb             T--NYCTKPQKSYLVGFSV-----------    138
Mouse TSHb             T--NYCTKPQSFYLGGFSV-----------    138
Rainbow TSHb           GDGARCSKPLRHIYPYPGLNSYIHPN----    147
Zebrafish TSHba        SAGMRCSKPVHHLYPEEN--NYAQAYWDQYE    148
                            #

Human TSHb C105V mutant   T—NYVPNLRSLIW----------------    133
Zebrafish TSHba mutant    SAGNLQTCASSVPRGEQLRPSILGPV-----    143
```

▯: signal peptide
@: glycosylation site
*: cysteine knot motif for dimer interface
#: cysteine for intramolecular disulfide bridge

B

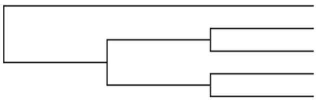

Zebrafish TSHbb 0.318005
Human TSHb 0.076087
Mouse TSHb 0.076087
Zebrafish TSHba 0.197279
Rainbow trout TSHb 0.197279

Figure S2

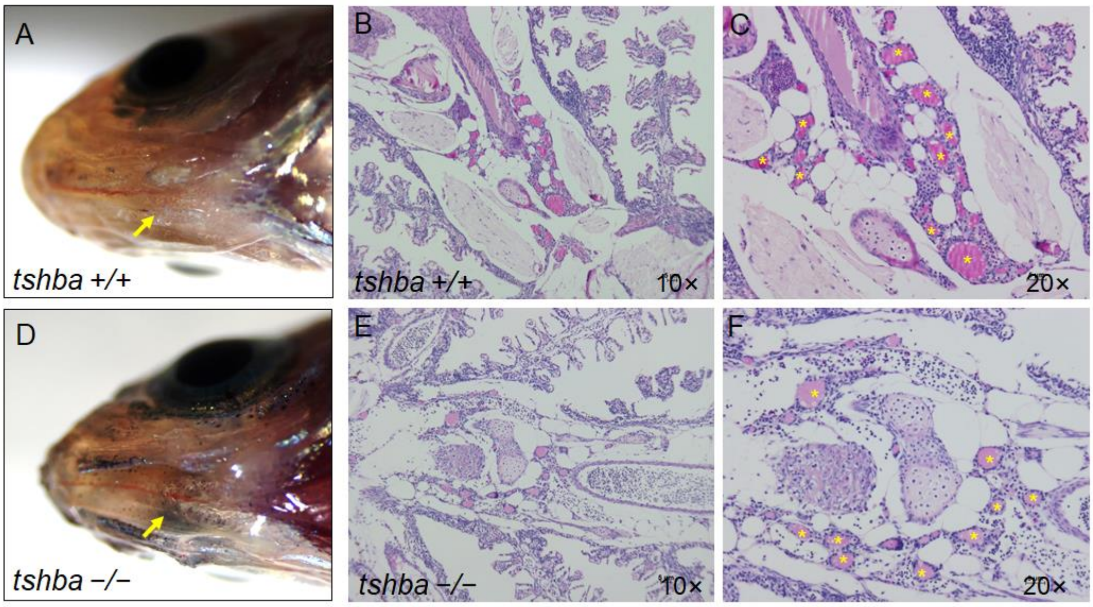

Figure S3

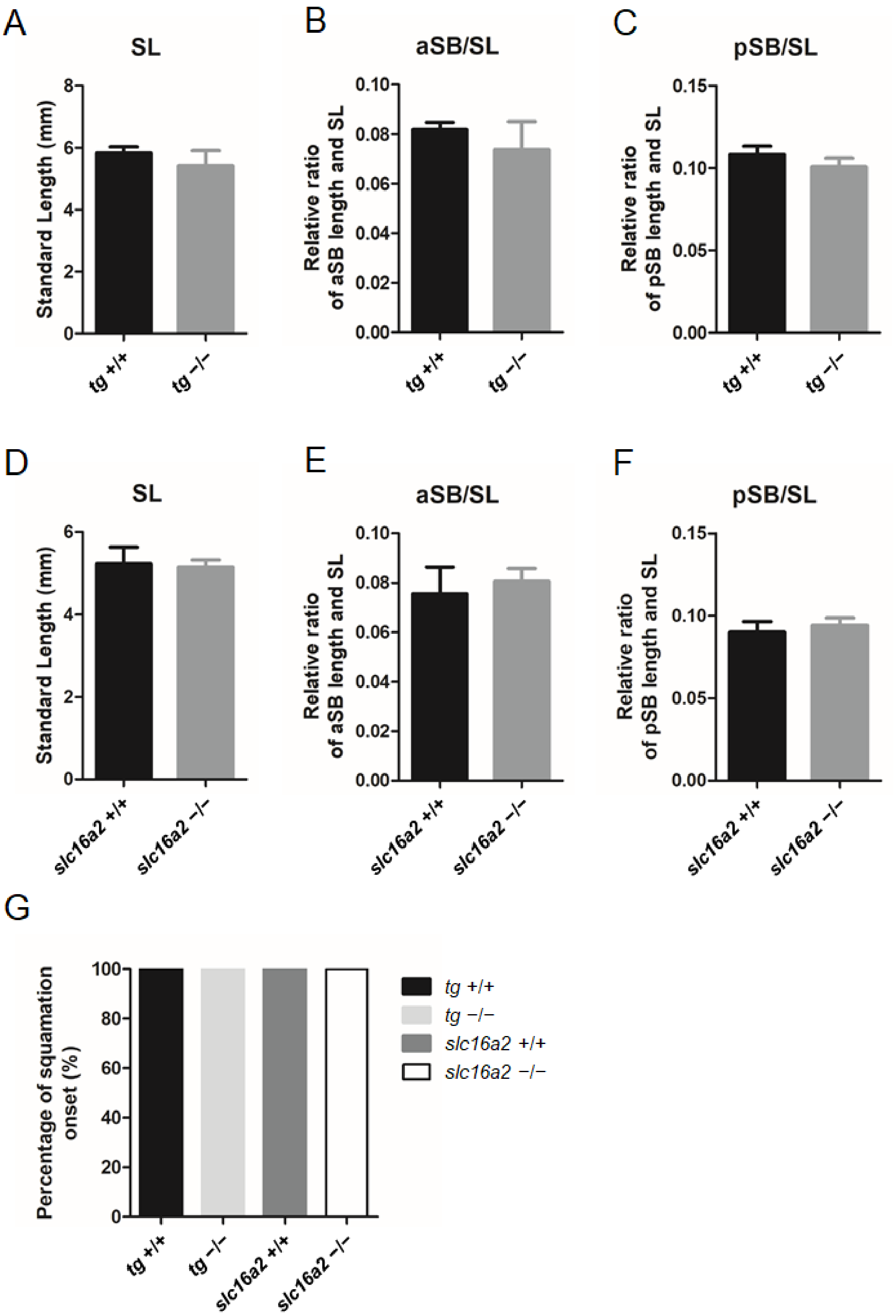

A
SL
Standard Length (mm)
8
6
4
2
0
tg +/+
tg −/−
B
aSB/SL
Relative ratio of aSB length and SL
0.10
0.08
0.06
0.04
0.02
0.00
tg +/+
tg −/−
C
pSB/SL
Relative ratio of pSB length and SL
0.15
0.10
0.05
0.00
tg +/+
tg −/−
D
SL
Standard Length (mm)
6
4
2
0
slc16a2 +/+
slc16a2 −/−
E
aSB/SL
Relative ratio of aSB length and SL
0.10
0.08
0.06
0.04
0.02
0.00
slc16a2 +/+
slc16a2 −/−
F
pSB/SL
Relative ratio of pSB length and SL
0.15
0.10
0.05
0.00
slc16a2 +/+
slc16a2 −/−
G
Percentage of squamation onset (%)
100
80
60
40
20
0
tg +/+
tg −/−
slc16a2 +/+
slc16a2 −/−
tg +/+
tg −/−
slc16a2 +/+
slc16a2 −/−

Figure S4

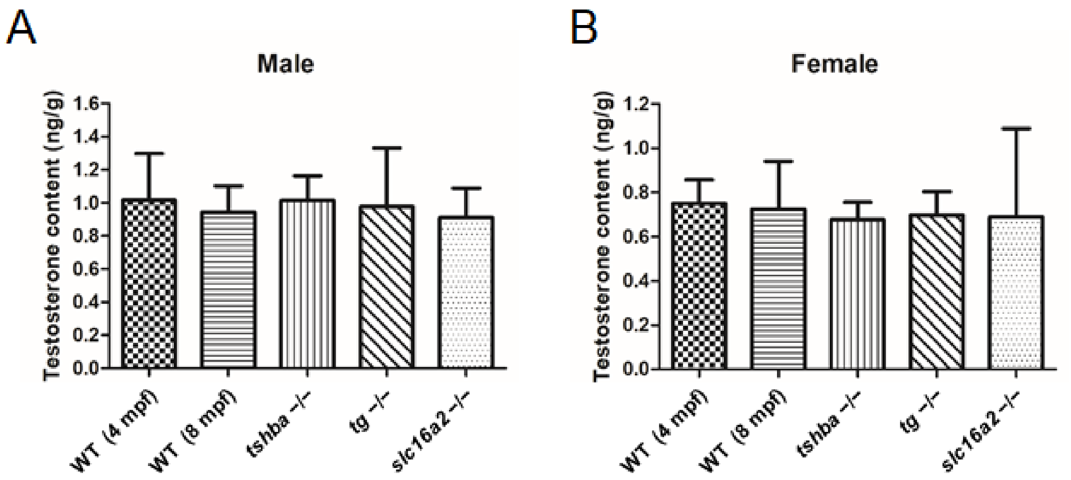
A
Male
Testosterone content (ng/g)
1.6
1.4
1.2
1.0
0.8
0.6
0.4
0.2
0.0
WT (4 mpf)
WT (8 mpf)
tshba −/−
tg −/−
slc16a2 −/−
B
Female
Testosterone content (ng/g)
1.2
1.0
0.8
0.6
0.4
0.2
0.0
WT (4 mpf)
WT (8 mpf)
tshba −/−
tg −/−
slc16a2 −/−

Supplementary tables

Table S1. Primers used for PCR reaction

Test	Gene Name	GenBank Accession Nos.	Sequences of Forward Primer (5'→3') and Reverse Primer (5'→3')		Product Length
Geno-typing	*tshba*	BX005150	Fw:	GATCCTCACTTCACCTACCCA	300 bp
			Rev:	CCAGTAACGTAATGGATTAGG	
	tg	CR855311	Fw:	TGCAATGTTTGATCACAAGTG	473 bp
			Rev:	GCATCCAGGGAAATGGATAA	
	slc16a2	NW_003335266	Fw:	CACCTCAGAGAACGTTTGCTT	623 bp
			Rev:	CAGATGGAAGATTCGACTCAC	
RT-PCR	*ef1α*	NM_131263.1	Fw:	GAGAAGTTCGAGAAGGAAGC	142 bp
			Rev:	CGTAGTATTTGCTGGTCTCG	
	tshba	NM_181494.2	Fw:	GGAGTGCAATTACTGTGTGG	139 bp
			Rev:	CCGGTACTCAACTTCCTGAT	
	tshbb	XM_017353619.2	Fw:	CACGTTGAGTTCAGTCTGGA	190 bp
			Rev:	CCAGCAGGAGCAGAAAACTG	
	tg	XM_689200.5	Fw:	CCAGCCGAAAGGATAGAGTTG	175 bp
			Rev:	ATGCTGCCGTGGAATAGGA	
	tpo	XM_009292775.1	Fw:	TGCCACGGACGAAGAATACC	183 bp
			Rev:	CGAACCGGAGGAAGTTGGAA	
	slc5a5	NM_001089391.1	Fw:	GCCACAGATTTCTGACACGC	204 bp
			Rev:	AAGACTGGAACAGCCCGATG	
	tg (full-length)	NM_001329865.1	Fw:	CCTCCTCAGTGTTCAGATGA	198 bp
			Rev:	CATCTCTCTTCCACGACTGT	
	tg (short)	none	Fw:	GCTTCAGGCACTGGAGTGAA	205 bp
			Rev:	GCTCCAACTCTGGCAATGAC	
	slc16a10	NM_001080028.1	Fw:	CTTCTGCTCTCCAATCGTCA	178 bp
			Rev:	CTGATACGCAAAACTGCAGC	
	oatp1c1	NM_001044997.3	Fw:	GCTGAACCCAGCACATCTTC	204 bp
			Rev:	GACGGAGATAGTCTGGACAC	
	slc16a2	NM_001258230.1	Fw:	GTGGTCAGTATGTTCACCGA	187 bp
			Rev:	GACCCAGGATCACCAGAGAT	
	cga	XM_005169876.4	Fw:	CACATCAGAAGCCACTTGCT	231 bp
			Rev:	CGAATTGGAAATGGCCACAG	

Continued table S1

Test	Gene Name	GenBank Accession Nos.	Sequences of Forward Primer (5′→3′) and Reverse Primer (5′→3′)		Product Length
RT-PCR	*pomca*	NM_181438.3	Fw:	GACCTCAGCACAGAGGAGAA	139 bp
			Rev:	GGTGTGCGAGGAGGTCGATT	
	prl	NM_181437.3	Fw:	CACCATTAACAGCAAGACCA	153 bp
			Rev:	CGAGACGTTTTATCCTGACC	
	lhb	NM_205622.2	Fw:	GCAGAGACACTTACAACAGCC	125 bp
			Rev:	AAAACCAAGCTCTGAGCAGCC	
	fshb	NM_205624.1	Fw:	GATGCGTGTGCTTGTTCTGG	177 bp
			Rev:	ACTCGATCCATTGTCCAGCAT	
	gh1	NM_001020492.2	Fw:	CAGCGGCTCTTCAACAACGC	113 bp
			Rev:	CTCAACTGTCTGCGTTCCTC	
	smtla	XM_021467699.1	Fw:	TGGTTCAGTCGTGGATGG	187 bp
			Rev:	AAGATGGTGGAGGATGCC	
	smtlb	NM_001037674.1	Fw:	TCTCGGAGGAAGCCAAGTTG	151 bp
			Rev:	AGCCATCGGTCGGAAATCTG	

Table S2. The primers used for the amplification of *tg* alternative short transcripts

Gene Name	GenBank Accession Nos.	Sequences of Forward Primer (5′→3′) and Reverse Primer (5′→3′)		Product Length
tg	NM_001329865.1	Fw:	CCAGCACACTGATTTGTATT	Full-length transcripts: 1136 bp<br>Short transcripts: 254 bp
		Rev:	GGAGAACACGTTGCTATTGC	
